# A Depolarizing Leak in Sodium Bicarbonate Cotransporter NBCe1 Causes Brain Edema

**DOI:** 10.1002/acn3.70363

**Published:** 2026-03-17

**Authors:** Quinty Bisseling, Mark D. Parker, Sven Kerst, Richard A. Pasternack, Jacob Tondreau, Marjolein Breur, Gemma M. van Rooijen‐van Leeuwen, Davide Tonduti, Ettore Salsano, Alejandra Darling, Joanna A. E. van Wijk, Susanna Törnroth‐Horsefield, Marianna Bugiani, Petra J. W. Pouwels, Quinten Waisfisz, Marjo S. van der Knaap, Rogier Min

**Affiliations:** ^1^ Department of Child Neurology, Amsterdam Leukodystrophy Center, Emma Children's Hospital Amsterdam University Medical Center, Amsterdam Neuroscience Amsterdam the Netherlands; ^2^ Department of Integrative Neurophysiology, Center for Neurogenomics and Cognitive Research Vrije Universiteit Amsterdam, Amsterdam Neuroscience Amsterdam the Netherlands; ^3^ Department of Physiology and Biophysics, Jacobs School of Medicine and Biomedical Sciences The State University of New York: The University at Buffalo Buffalo New York USA; ^4^ Department of Ophthalmology, Jacobs School of Medicine and Biomedical Sciences The State University of New York: The University at Buffalo Buffalo New York USA; ^5^ Unit of Pediatric Neurology, C.O.A.L.A (Center for Diagnosis and Treatment of Leukodystrophies) Vittore Buzzi Children's Hospital Milan Italy; ^6^ Department of Biomedical and Clinical Sciences Università Degli Studi di Milano Milan Italy; ^7^ Unit of Rare Neurological Diseases Fondazione IRCCS Istituto Neurologico Carlo Besta Milan Italy; ^8^ Neurometabolic Unit, Movement Disorders Unit, Neurology Department Hospital Sant Joan de Déu Barcelona Spain; ^9^ Department of Pediatric Nephrology Amsterdam University Medical Center Amsterdam the Netherlands; ^10^ Department of Biochemistry and Structural Biology Lund University Lund Sweden; ^11^ Department of Pathology, Amsterdam Leukodystrophy Center Amsterdam University Medical Center, Amsterdam Neuroscience Amsterdam the Netherlands; ^12^ Department of Radiology and Nuclear Medicine Amsterdam University Medical Center, Amsterdam Neuroscience Amsterdam the Netherlands; ^13^ Department of Human Genetics Amsterdam University Medical Center Amsterdam the Netherlands

**Keywords:** astrocyte, brain edema, leukodystrophy, NBCe1, renal tubular acidosis, *SLC4A4*

## Abstract

**Objectives:**

*SLC4A4* encodes electrogenic sodium bicarbonate cotransporter NBCe1, prominently expressed in kidney and brain. Recessive loss‐of‐function variants in *SLC4A4* cause proximal renal tubular acidosis, no brain edema. In the brain, NBCe1 is expressed by astrocytes, where it regulates pH and mediates astrocyte volume changes. Here we describe a novel dominant variant in *SLC4A4* in patients with brain edema and investigate how it affects NBCe1 function.

**Methods:**

Genetic studies identified a novel gene variant in three unrelated pediatric patients with the same MRI pattern of cerebral subcortical white matter signal abnormality and swelling, and medulla lesions. Immunohistochemical and electrophysiological experiments were performed to determine the localization of the transporter in the brain and the functional consequence of the patient variant.

**Results:**

The same heterozygous variant in *SLC4A4* was found in all three patients and one parent. The children displayed infantile‐onset progressive macrocephaly, motor and cognitive impairment, autism, epilepsy, and recurrent episodes of increased intracranial pressure. Bicarbonate treatment of two patients led to clinical and MRI improvement. Immunohistochemistry revealed that brain NBCe1 is mainly present in astrocytes, more in cortex than white matter. Functional experiments revealed impaired transporter activity of mutant NBCe1 due to reduced membrane expression and a prominent depolarizing ion leak.

**Interpretation:**

The most likely pathomechanism of this novel *SLC4A4*‐related disease is that a depolarizing leak in NBCe1 disrupts astrocyte pH regulation, promoting swelling and impairing volume control. These findings uncover a previously unrecognized mechanism of genetic brain edema and establish NBCe1 as a critical modulator of astrocyte homeostasis.

**Abbreviations**
AD: axial diffusivity
AE1: chloride bicarbonate anion exchanger 1
CD68: cluster of differentiation 68
DWI: diffusion‐weighted imaging
FA: fractional anisotropy
GFAP: glial fibrillary acidic protein
IRBIT: IP3R‐binding protein released with inositol 1,4,5‐trisphosphate
MAP2: microtubule‐associated protein 2
MD: mean diffusivity
MLC: megalencephalic leukoencephalopathy with subcortical cysts
NBCe1: electrogenic sodium bicarbonate cotransporter 1
NDCBE: sodium‐driven chloride/bicarbonate exchanger
PAE: predicted alignment error
pLDDT: predicted local distance difference test
pRTA: proximal renal tubular acidosis
RD: radial diffusivity
ROI: region‐of‐interest
*SLC4A4*
: solute carrier family 4 member 4
SNP: single nucleotide polymorphism
V_eq_
: reversal potential
WES: whole exome sequencing
WGA: wheat germ agglutinin

## 1 Introduction

Brain edema is a potentially life‐threatening consequence of neurological injury and disease. Tight control over brain ion and water dynamics is therefore crucial. Astrocytes are central in this [1]. They express a wide array of channels, pumps, transporters, and structural proteins that together maintain osmotic balance and regulate ion and water distribution across brain compartments. Disruption of this machinery through genetic defects can lead to disorders characterized by chronic brain edema [2]. The best‐characterized example is megalencephalic leukoencephalopathy with subcortical cysts (MLC) [3], a rare leukodystrophy caused by genetic defects affecting astrocyte proteins MLC1 [4], GlialCAM [5], GPRC5B, or AQP4 [6]. Studying MLC and related monogenic diseases has advanced molecular understanding of brain ion and water homeostasis and mechanisms that prevent edema.

While ions and water are clearly linked to brain volume regulation, a third component has received less attention: acid–base homeostasis. It is well known that lowering arterial CO_2_ through hyperventilation induces extracellular alkalosis, vasoconstriction, and reduction in intracranial pressure, underscoring the role of pH regulation in brain volume control [7]. Astrocytes are intimately involved in pH regulation [8], but the connection between astrocyte acid–base homeostasis and brain edema is poorly understood. No monogenic disorders have been linked to disrupted acid–base regulation as a driver of brain edema.

A key molecular player in acid–base regulation is the electrogenic sodium bicarbonate cotransporter NBCe1, encoded by *SLC4A4* [9]. NBCe1 enables astrocytes to control intracellular pH and buffer changes in extracellular acidity, particularly upon increased neuronal activity [10]. By coupling bicarbonate transport to sodium gradients, it serves as a link between pH homeostasis and ionic equilibrium. Activity‐dependent bicarbonate transport is involved in astrocyte swelling [11, 12], but the contribution of NBCe1 to brain volume control is unexplored.

Outside the brain, *SLC4A4* is highly expressed in other organs, including kidney, eye, and teeth [9]. In renal proximal tubules, NBCe1 reabsorbs filtered bicarbonate and is critical for maintaining systemic acid–base balance. Biallelic loss‐of‐function variants in *SLC4A4* cause proximal renal tubular acidosis (pRTA), a disorder characterized by systemic acidosis, short stature, ocular abnormalities, dental enamel defects, and occasionally neurological symptoms such as intellectual disability and epilepsy [13, 14, 15]. However, chronic brain edema does not occur in pRTA.

In this study, we identify a novel heterozygous variant in *SLC4A4* in pediatric patients presenting with chronic and episodic brain edema and shared clinical and neuroimaging features.

## 2 Methods

Detailed methods are available as Supporting Information.

### 2.1 Patients, Genetics, and Laboratory Findings

We identified three patients with infantile‐onset macrocephaly sharing the same MRI pattern of edema of subcortical cerebral white matter and medulla lesions. We performed trio‐whole exome sequencing (trio‐WES) on genomic DNA from two unrelated patients, patients 1 and 2, and their parents. We analyzed patient 3 and his mother for the gene variant identified in patients 1 and 2 by Sanger sequencing. Laboratory investigations focused on pRTA were done in patients 1 and 2.

### 2.2 MRI

T1‐weighted, T2‐weighted, and FLAIR sequences were available for all patients. In patients 1 and 2, sequential MRI included volumetric analysis of 3D structural images and extraction of quantitative measures based on diffusion‐weighted imaging (DWI) from selected regions‐of‐interest (ROIs), as described [16].

### 2.3 Western Blot, Immunohistochemistry, and Immunofluorescence

NBCe1 isoform expression was studied using western blot on gray and white matter lysates prepared from postmortem human brain samples. Immunohistochemistry and immunofluorescence staining on human brain tissue samples were used to study cell‐type and subcellular distribution of NBCe1 with a pan‐isoform antibody.

### 2.4 Cellular and Molecular Studies

HEK293 cells transiently transfected with eGFP‐tagged human NBCe1‐B were used for whole cell patch‐clamp analysis and confocal imaging. *Xenopus laevis* oocytes, injected with cRNA encoding human NBCe1‐B, sometimes together with IRBIT, were used for two‐electrode voltage clamp analysis and cell surface biotinylation.

### 2.5 Structural Modeling Using AlphaFold

Structures of wild‐type and mutant NBCe1‐B were predicted using AlphaFold 3 [17]. Local and global confidence merits were evaluated based on predicted Local Distance Difference Test (pLDDT) and Predicted Alignment Error (PAE). Substrate positions were modeled from their position in the experimental structure of sodium‐driven chloride/bicarbonate exchanger NDCBE (PDB code 7RTM). Diameter of the substrate‐conducting pathway was calculated using HOLE [18].

### 2.6 Statistics

HEK293 cell data were tested for normality with a Kolmogorov–Smirnov test. Depending on normality, data were compared using unpaired *t*‐test or Mann–Whitney test. For oocyte data, multiple groups were compared using a General Linear Model, followed by Tukey's method for group comparisons. Statistically significant differences were defined as *p* ≤ 0.05. Data are represented as mean ± SEM.

### 2.7 Study Approval

Patient data were reviewed with the approval of the Ethics Committee and written informed consent from the parents. Methods for harvesting ovaries from *Xenopus laevis* frogs were approved by the Institutional Animal Care and Use Committee.

## 3 Results

**FIGURE 1:**
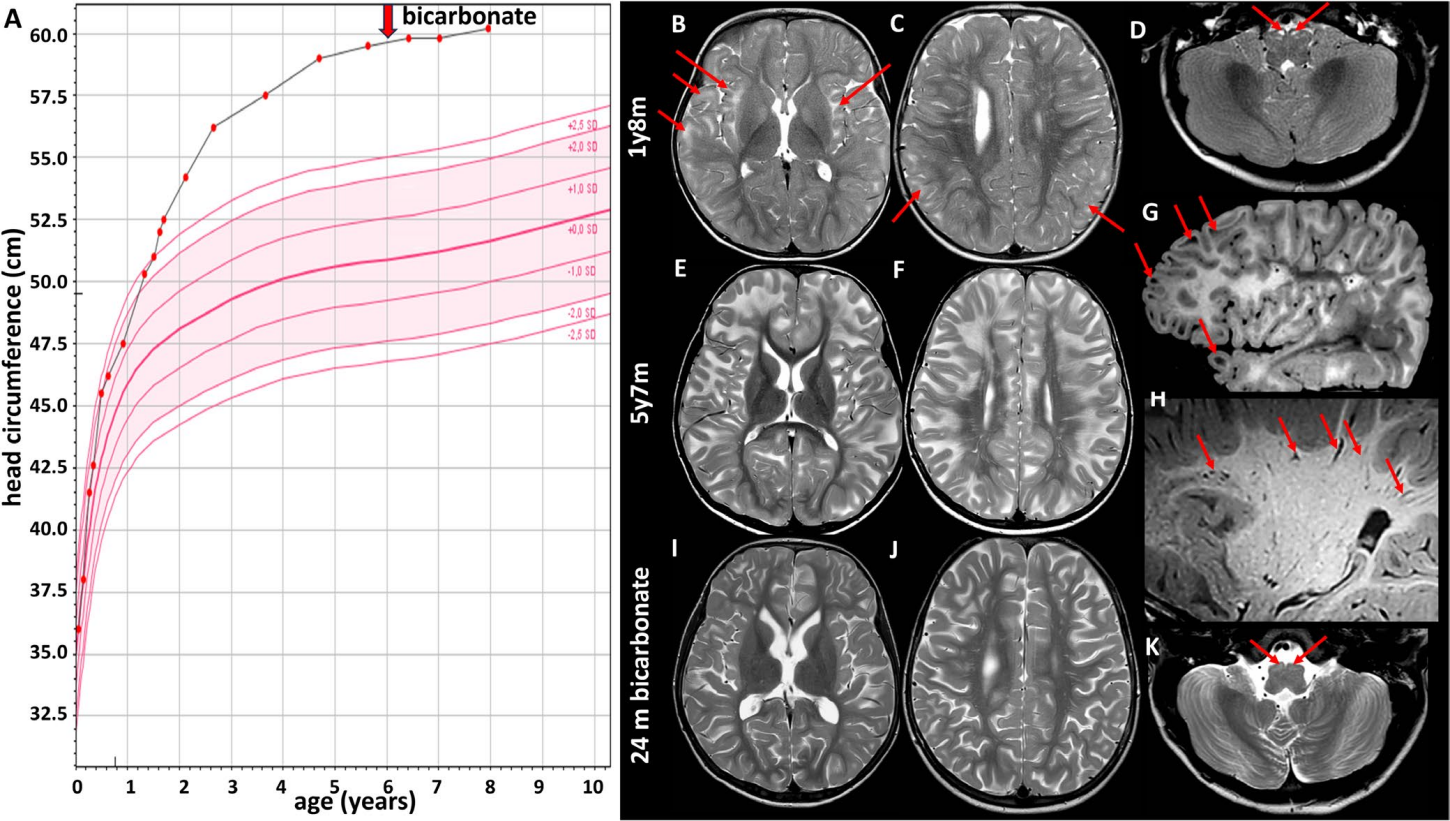
Brain MRI and head circumference in patient 1. (A) Head circumference. The red arrow indicates the start of bicarbonate treatment. (B–D) The first MRI at 1y8m shows minor abnormalities restricted to the directly subcortical cerebral white matter (arrows in B, C) and signal abnormalities in the pyramids and hilum of the inferior olives in the medulla (arrows in D). (E–H) Follow‐up MRI at 5y7m shows an increase in extensiveness and degree of swelling of the cerebral white matter abnormalities, with a decreasing zone of normal periventricular white matter (E, F). Especially, the directly subcortical white matter is rarefied to cystic (arrows in G, also note the anterior temporal cyst). A radiating pattern of enlarged perivascular spaces is seen in the cerebral white matter (arrows in H). (I–K) MRI after 24 months treatment shows a marked decrease in the cerebral white matter edema (I, J), and resolution of the signal abnormalities in the medulla (arrows in K). y, years; m, months.

**FIGURE 2:**
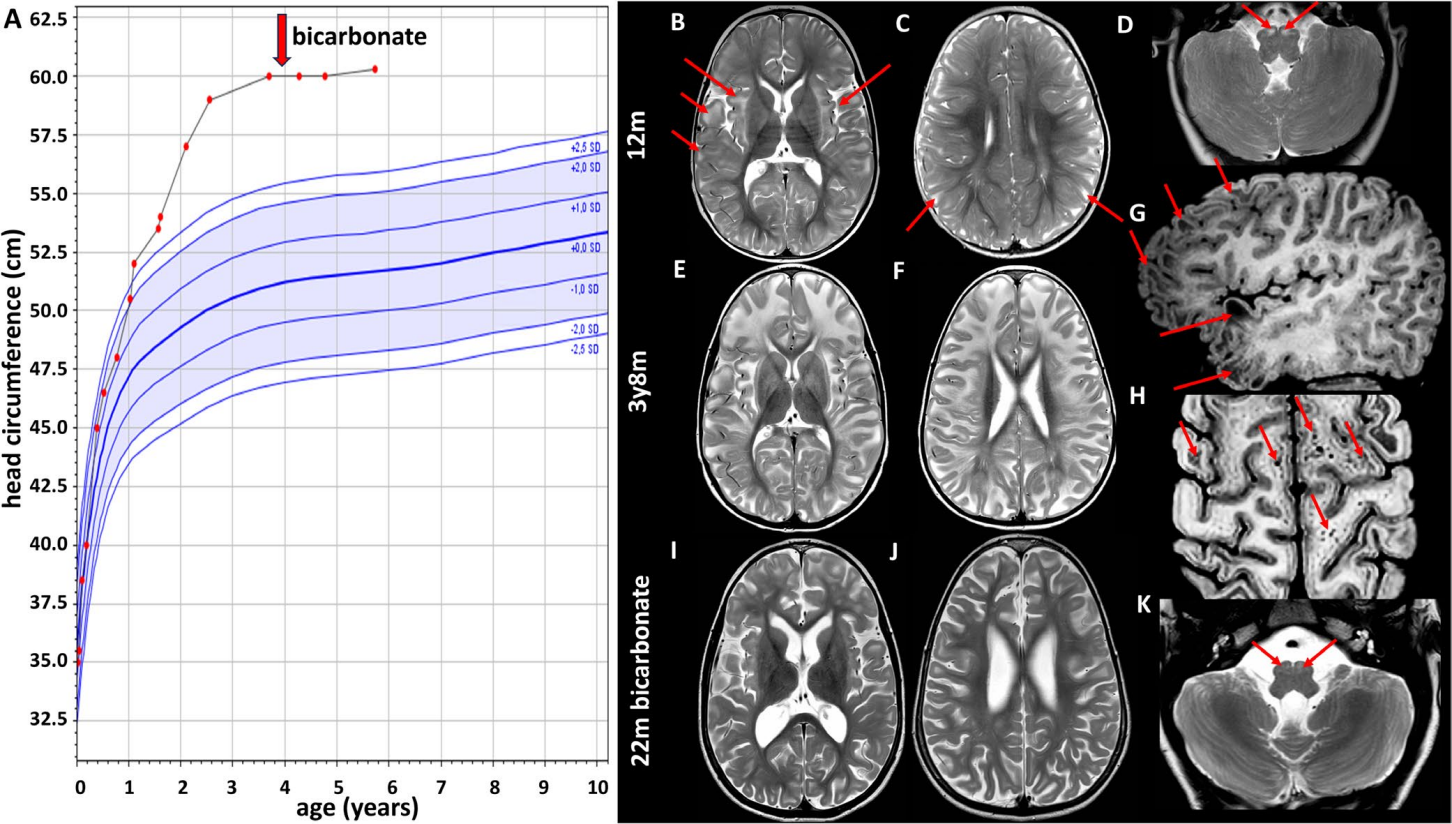
Brain MRI and head circumference in patient 2. (A) Head circumference. The red arrow indicates the start of bicarbonate treatment. (B–D) The first MRI at 12m shows minor abnormalities restricted to the directly subcortical cerebral white matter (arrows in B, C) and signal abnormalities in the pyramids and hilum of the inferior olives in the medulla (arrows in D). (E–H) Follow‐up MRI at 3y8m shows an increase in extensiveness and degree of swelling of the cerebral white matter abnormalities, with a decreasing zone of normal periventricular white matter (E, F). Especially, the directly subcortical white matter is rarefied to cystic (arrows in G; also note the anterior temporal cyst). A pattern of enlarged perivascular spaces is seen in the cerebral white matter (arrows in H). (I–K) MRI after 22 months treatment shows a marked decrease in the cerebral white matter edema (I, J) and resolution of the signal abnormalities in the medulla (arrows in K). y, years; m, months.

We identified three unrelated children (patients 1–3) with infantile‐onset macrocephaly and the same MRI pattern of edema of the subcortical cerebral white matter and medulla lesions (Figures 1 and 2). Genetic studies revealed that all three share the same heterozygous missense variant in *SLC4A4* (c.2414T>C in NM_001098484.3; c.2282T>C in NM_003759.4), encoding electrogenic sodium bicarbonate cotransporter NBCe1. Alternative splicing can yield three NBCe1 isoforms, NBCe1‐A, ‐B and ‐C [9]. The missense variant results in a non‐conservative amino acid substitution (p.(Ile805Thr) in NBCe1‐B and p.(Ile761Thr) in NBCe1‐A). This amino acid is highly conserved among SLC4 proteins; the variant is absent from gnomAD v4.1.0 (gnomad.broadinstitute.org). In patients 1 and 2, the variant was absent in parents, suggesting it arose *de novo*. Patient 3 inherited the variant from his mother (referred to as patient 4).

### 3.1 Clinical Phenotype Before Treatment

Patients 1–3 presented with progressive macrocephaly in the second half of the first year, crossing the +2SD at the end of the first year, with head circumferences rising to > +5SD by early childhood (Figures 1A and 2A). In patient 1, development was delayed from early on. Patients 2 and 3 presented at approximately 12 months of age with neurological regression and signs of increased intracranial pressure. Over the years, all had recurrent episodes of raised intracranial pressure, provoked by minor head trauma or intercurrent infections, responsive to treatment with corticosteroids (patients 2 and 3) and acetazolamide (patient 2). They had prominent cerebellar ataxia; patient 3 also had spasticity. Patient 1 achieved unsupported but unstable walking; patients 2 and 3 could not sit without support. All three had autistic features and no language. They developed seizures, which were controlled by anticonvulsants. Patient 3 died at age 20 years due to a respiratory infection. Patient 4 experienced minor head trauma from a fall at age 44 years. Subsequently, she developed persistent headaches, mood fluctuations, and gait difficulties. After another minor head trauma, she experienced further decline with increasing gait instability and dysarthria. At age 52 years, she resides in a neurological disability center. None of the patients exhibited overt signs of pRTA. They had normal stature; dentition and enamel were unremarkable. In patients 1 and 2, laboratory investigations were performed, which revealed mild metabolic acidosis with slightly reduced serum [HCO_3_^−^] and a normal anion gap, consistent with mild pRTA ([HCO_3_^−^] (mmol/L) patient 1: 19.6; patient 2: 21.3; reference: 23–29; Table S5).

### 3.2 MRI Abnormalities and Quantitative MRI Analysis Before Treatment

Patient 1 underwent 5 MRIs between ages 1 year 8 months (1y8m) and 5y7m (Figure 1); patient 2 underwent 4 MRIs between 12m and 3y8m (Figure 2); and patient 3 underwent 4 MRIs between 1y4m and 4y7m (Figure S1), revealing the same pattern and sequence of abnormalities. Initially, minor signal abnormalities with slight swelling were observed in the directly subcortical cerebral white matter, which over time increased in extensiveness and degree of swelling. Small cysts were present in the anterior temporal region. Additionally, signal abnormalities in the pyramids and hilum of the inferior olives in the medulla were consistently present. In patients 1 and 2, with higher resolution MRIs, dilated perivascular spaces were striking. Patient 3 also had signal abnormalities in the cerebellar subcortical white matter and pyramidal tracts in midbrain and pons.

In patient 3, follow‐up MRIs at 6y9m and 7y2m (Figure S1) showed decreased cerebral white matter abnormalities and swelling, accompanied by cerebral atrophy, but medulla abnormalities remained unchanged. Patient 4 underwent MRIs at ages 45y (Figure S1) and 52y, which showed no signal abnormalities, but marked cerebral and cerebellar atrophy.

**FIGURE 3:**
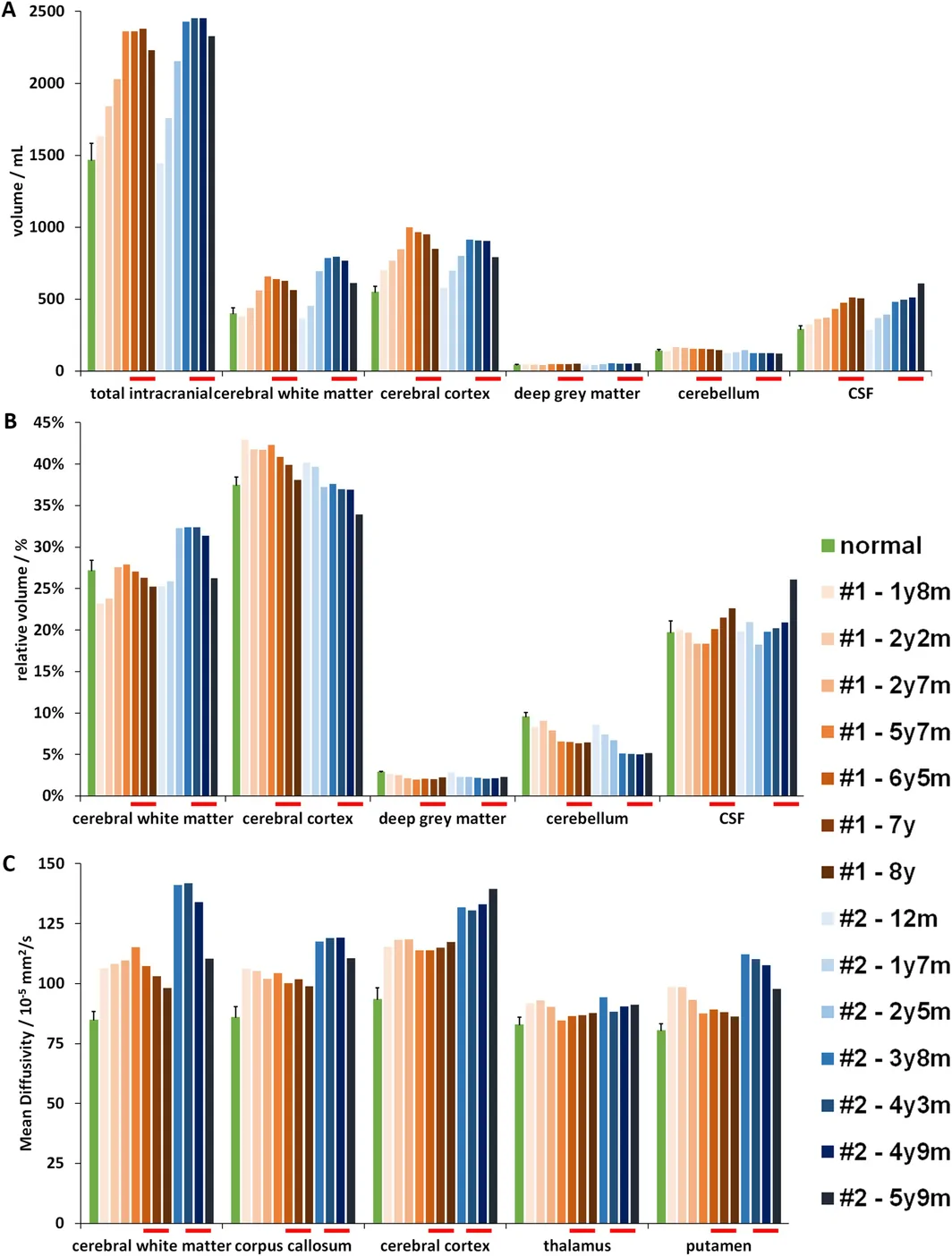
Brain MRI volumetry and diffusion parameters. (A) Total intracranial volume and volumes of cerebral white matter, cerebral cortex, deep gray matter, cerebellum and cerebrospinal fluid (CSF) of patient 1 (shades of brown) and patient 2 (shades of blue). Normal age‐matched values are shown in green, with the error bar representing 1SD. Red lines under the *X*‐axis indicate bicarbonate treatment. (B) Relative volumes normalized by the intracranial volume. (C) Quantitative values of mean diffusivity (MD) in several white matter and gray matter regions‐of‐interest (ROIs) (for patient 2 no diffusion data were available between ages 12m and 2y5m). Volumes of other structures and other diffusion measures are presented in Tables S1–S4. Deep gray matter comprises thalamus, caudate nucleus, putamen, pallidum, amygdala and nucleus accumbens. y, years; m, months.

We performed quantitative MRI volumetric measurements on the MRIs of patients 1 and 2 (Figure 3, Tables S1 and S2). In patient 1, total intracranial volume was high‐normal for age at 1y8m and continued to increase (Figure 3A). In patient 2, intracranial volume was normal at 12m and strongly increased after 2y. Volumes of cerebral white matter, cerebral cortex, and CSF showed corresponding increases. Volumes of combined deep gray matter structures and cerebellum were normal or high‐normal. When expressed as percentage of total intracranial volume (Figure 3B), relative cerebral cortex volume was increased at all ages in patient 1, and at early ages (12m and 1y7m) in patient 2. Relative cerebral white matter volume was reduced at early ages (1y8m and 2y2m) and normal from 2y7m in patient 1, while initially normal and elevated from 2y5m in patient 2. Relative CSF volume was normal, while deep gray matter and cerebellar volumes were reduced. These findings indicate an initial increase in cerebral cortex volume, while cerebral white matter volume increased later.

Single‐shell DWI revealed abnormal measures in most brain structures. In patient 1 after age 2y7m, and in patient 2 after age 3y8m, fractional anisotropy (FA) was strongly decreased in cerebral white matter and corpus callosum (Table S3). FA was normal in thalamus and most other deep gray matter structures, and in cerebral cortex only reduced at older ages (Table S4). Mean diffusivity (MD), axial diffusivity (AD), and radial diffusivity (RD) were generally strongly increased in cerebral cortex, cerebral white matter, and corpus callosum (Figure 3C). Diffusivity values in deep gray matter structures were generally increased, but to a lesser degree. These widespread DWI changes are in line with diffuse edema of mainly cerebral cortex and white matter.

### 3.3 Clinical and MRI Improvement With Bicarbonate Treatment

In patients with pRTA, treatment with bicarbonate is critical to limit clinical consequences of acidosis. We investigated whether bicarbonate treatment would benefit patients with *SLC4A4*‐related encephalopathy. From age 6y, patient 1 was treated with oral bicarbonate 3 daily doses (3dd) 20 mmol at a weight of 28 kg (Table S5), while patient 2 was treated from age 4y with oral bicarbonate 2dd 36 mmol at a weight of 23 kg (Table S5). From treatment onset, their disposition markedly changed with improved contact, interaction, display of emotions and interest in their surroundings. In the 24 months (patient 1) and 22 months (patient 2) of treatment, ataxia decreased and gross and fine motor performances improved. Patient 1 learned to walk stairs, while patient 2 could sit without support and crawl. For both the increase in head circumference normalized under treatment (Figures 1A and 2A).

After 6, 12 and 24 months treatment for patient 1, and 4, 10 and 22 months for patient 2, MRI showed decreasing cerebral white matter edema (Figures 1I,J and 2I,J) and resolution of signal abnormalities in the medulla (Figures 1K and 2K). In both patients, sequential quantitative MRI demonstrated that from the start of treatment, absolute and relative cerebral white matter and cerebral cortex volumes no longer showed a continuous increase, but a stabilization or slight decrease, with a small increase in CSF volume (Figure 3A,B; Tables S1 and S2). Diffusion parameters improved on MRI follow‐up in both patients, with decreasing AD, MD and RD, particularly in cerebral white matter (Figure 3C; Tables S3 and S4). In conclusion, treatment with oral bicarbonate led to clear clinical improvement, stabilization of head circumference, and amelioration of morphologic and quantitative MRI abnormalities.

### 3.4 NBCe1 Expression in Astrocytes in the Human Brain

**FIGURE 4:**
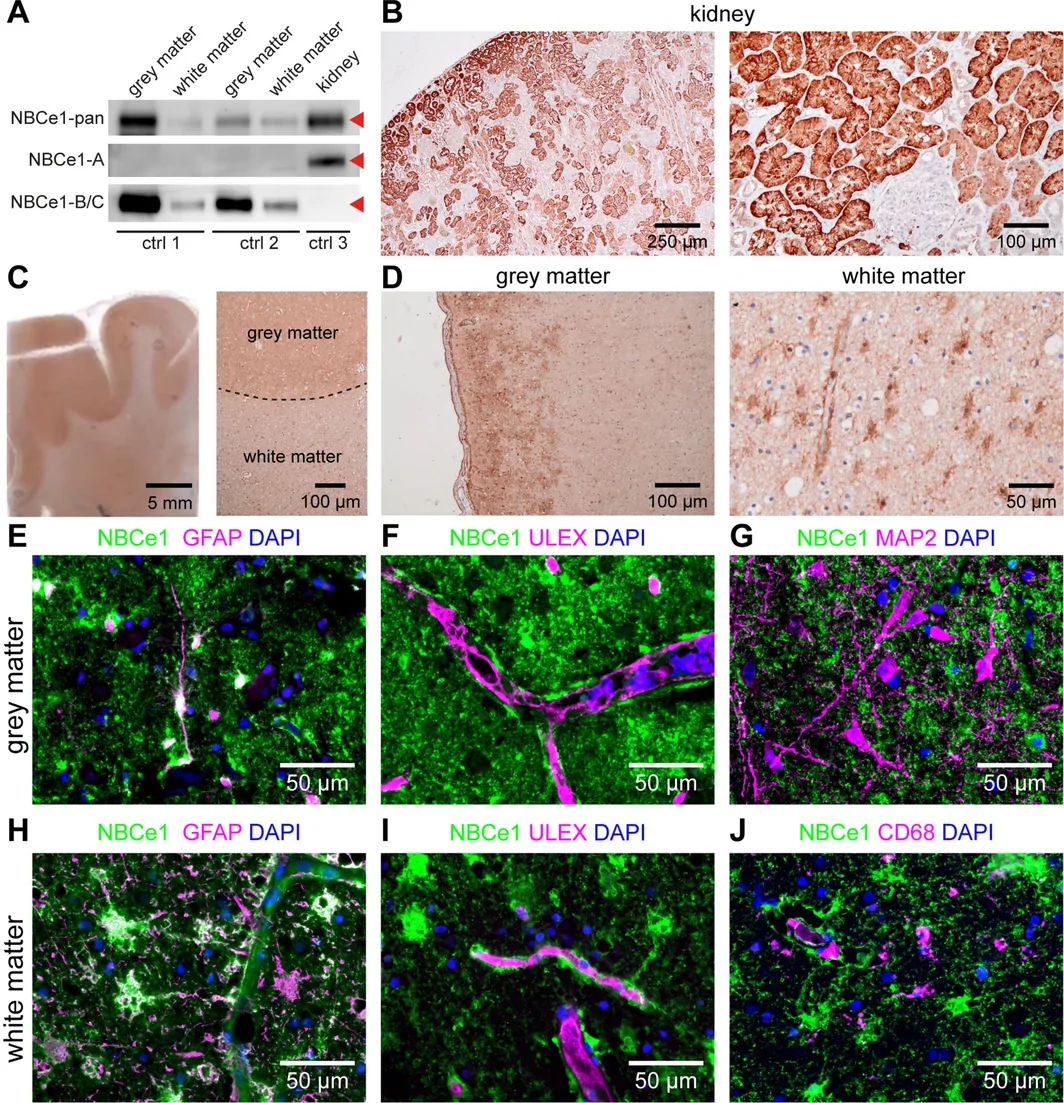
NBCe1 expression and localization in the human brain. (A) Western blots from human gray and white matter lysates from two different subjects and a kidney lysate from a third subject, using either an antibody recognizing all NBCe1 isoforms (NBCe1‐pan; top) or only the NBCe1‐A (middle) or NBCe1‐B/C (bottom) isoforms. Red arrow indicates the expected band for NBCe1 protein (~130 kDa). NBCe1‐B/C is the main brain isoform of the transporter, while NBCe1‐A is mainly expressed in kidney. Full blots shown in Figure S3. (B–D) Immunohistochemical (DAB) staining of human tissue using the NBCe1‐pan antibody. In kidney, prominent labeling of proximal tubules is observed (B). In human brain, both white and gray matter are positive (C), but gray matter staining is more pronounced. Higher magnification reveals clear staining of bushy cells (astrocytes), as well as enrichment of staining at the pial surface and in layer 1 astrocytes (left) and surrounding vessels (right; D). (E–J) Immunofluorescence staining using NBCe1‐pan (green) reveals a punctate staining pattern throughout the neuropil that resembles the bushy morphology of astrocytes. Co‐labeling with the astrocyte marker GFAP (magenta) confirms astrocytic origin of the signal in gray and white matter (E, H). Labeling of blood vessels with ULEX (magenta) reveals clear NBCe1 positivity of perivascular astrocyte endfeet in gray and white matter (F, I). MAP2‐positive neurons (magenta) in gray matter do not show NBCe1 positivity (G). No immunoreactivity is observed in CD68‐positive microglia (magenta) in white matter (J).

*SLC4A4* is expressed in different organs, with enrichment in kidneys and brain (Figure S2A). Public RNA expression databases show *SLC4A4* RNA throughout the brain, mainly in mature astrocytes (Figure S2B,C). Previous studies suggest NBCe1‐B as main brain isoform [19, 20], but data from human tissue is scarce. We performed western blot analysis on gray and white matter lysates (Figure 4A, Figure S3). A pan‐isoform antibody revealed NBCe1 presence in gray and white matter, while an antibody specific for NBCe1‐A [21] did not show brain presence. NBCe1‐B/C expression, studied with a recently validated antibody that binds a shared B/C‐epitope [21], revealed presence in white and gray matter, highest in gray, confirming that NBCe1‐B/C is the main brain isoform.

Immunohistochemistry and immunofluorescence microscopy with the pan‐isoform antibody was performed to identify cell types expressing NBCe1‐B/C (Figure 4B–J). In human adult frontal tissue, staining was slightly higher in gray than in white matter (Figure 4C), most prominently in perivascular areas and glia limitans. The staining pattern suggests expression over astrocyte fine processes (Figure 4D). A similar pattern was observed in tissue from children, without obvious developmental changes (Figure S4). Staining was observed throughout the brain (Figure S5). In all regions, expression appeared astrocytic, with enrichment at brain‐fluid interfaces. Immunofluorescence revealed dense punctate staining in gray and white matter (Figure 4E–J), suggestive of staining of fine astrocyte branches. Co‐staining with glial fibrillary acidic protein (GFAP) confirmed astrocyte expression (Figure 4E,H). Perivascular astrocyte endfeet surrounding vessels, visualized using anti‐ulex europaeus lectin (ULEX), showed clear positivity (Figure 4F,I). No expression was observed in microtubule‐associated protein 2 (MAP2)‐positive neurons (Figure 4G) or cluster of differentiation 68 (CD68)‐positive microglia (Figure 4J).

### 3.5 A Depolarizing Ion Leak in Mutant NBCe1‐B

**FIGURE 5:**
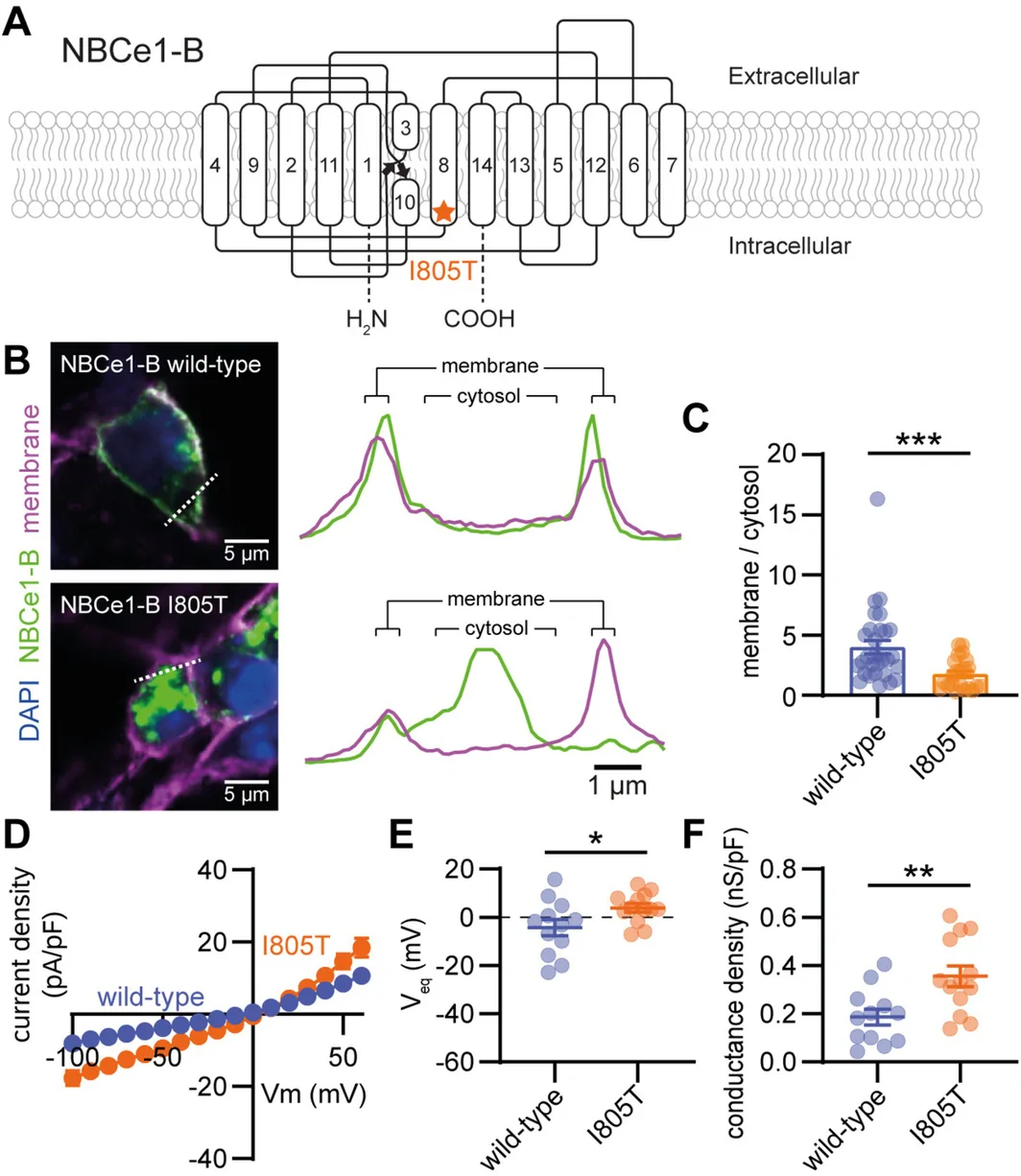
The NBCe1‐B p.(Ile805Thr) variant causes a depolarizing leak in transiently transfected HEK293 cells. (A) Schematic illustration of NBCe1‐B topology [22]. The location of the novel patient variant p.(Ile805Thr) is indicated by an orange star. (B) Left: Confocal imaging of HEK293 cells overexpressing eGFP‐tagged wild‐type or p.(Ile805Thr) human NBCe1‐B (green). Wild‐type NBCe1‐B co‐localizes with a fluorescent membrane marker (wheat germ agglutinin (WGA); magenta), indicating membrane expression. Membrane expression is reduced for p.(Ile805Thr) NBCe1‐B. Right: Fluorescence intensity profiles along dotted lines drawn left. (C) Wild‐type NBCe1‐B shows significantly more membrane localization compared to p.(Ile805Thr) NBCe1‐B (wild‐type: 3.99 ± 0.56, *n* = 30; p.(Ile805Thr): 1.74 ± 1.28, *n* = 22; *p* = 0.0003). (D) Averaged I/V curve from HEK293 cells transfected with wild‐type (blue) or p.(Ile805Thr) (orange) NBCe1‐B. (E) Bar graph showing the mean reversal potential (V_eq_) for I/V curves obtained from transfected HEK293 cells. p.(Ile805Thr)‐transfected HEK293 cells show a significantly depolarized V_eq_ (wild‐type: −4.29 ± 3.32 mV, *n* = 12; p.(Ile805Thr): 3.91 ± 1.72 mV, *n* = 13; *p* = 0.044). (F) Bar graph showing conductance density measurements (slope of the I/V curve between −20 and +20 mV divided by cell capacitance) from transfected HEK293 cells. p.(Ile805Thr)‐transfected HEK293 cells show a significantly higher conductance density (wild‐type: −0.18 ± 0.06 nS/pF, *n* = 12; p.(Ile805Thr): 0.36 ± 0.04 nS/pF, *n* = 13; *p* = 0.005). Dots in bar graphs and scatter plots indicate individual values. Data are presented as mean ± SEM. Asterisks indicate statistical significance (*p < 0.05, **p < 0.01, ***p < 0.001).

The p.(Ile805Thr) variant affects a residue in the eighth transmembrane domain of NBCe1 (Figure 5A), located just below the ion coordination site of the transporter [22]. Confocal imaging of HEK293 cells transiently transfected with the NBCe1‐B isoform of human *SLC4A4* carrying a C‐terminal eGFP tag [15] revealed strongly reduced membrane expression of p.(Ile805Thr) compared to wild‐type (Figure 5B,C).

Whole cell patch‐clamp recordings from transiently transfected HEK293 cells, performed in nominally HCO_3_^−^‐free conditions, revealed a constitutive depolarizing leak current in cells transfected with p.(Ile805Thr)‐NBCe1‐B compared to cells transfected with wild‐type NBCe1‐B (Figure 5D–F). This leak was reflected both in a significantly depolarized reversal potential (V_eq_) for the I/V curve of p.(Ile805Thr)‐transfected cells compared to wild‐type (Figure 5D,E), as well as in an increased slope of the current density versus membrane voltage curve (conductance density; Figure 5F). Transport activity of NBCe1‐B is inhibited by an auto‐inhibitory domain present in the protein isoform, and could therefore not be assessed in HEK293 cells [23].

**FIGURE 6:**
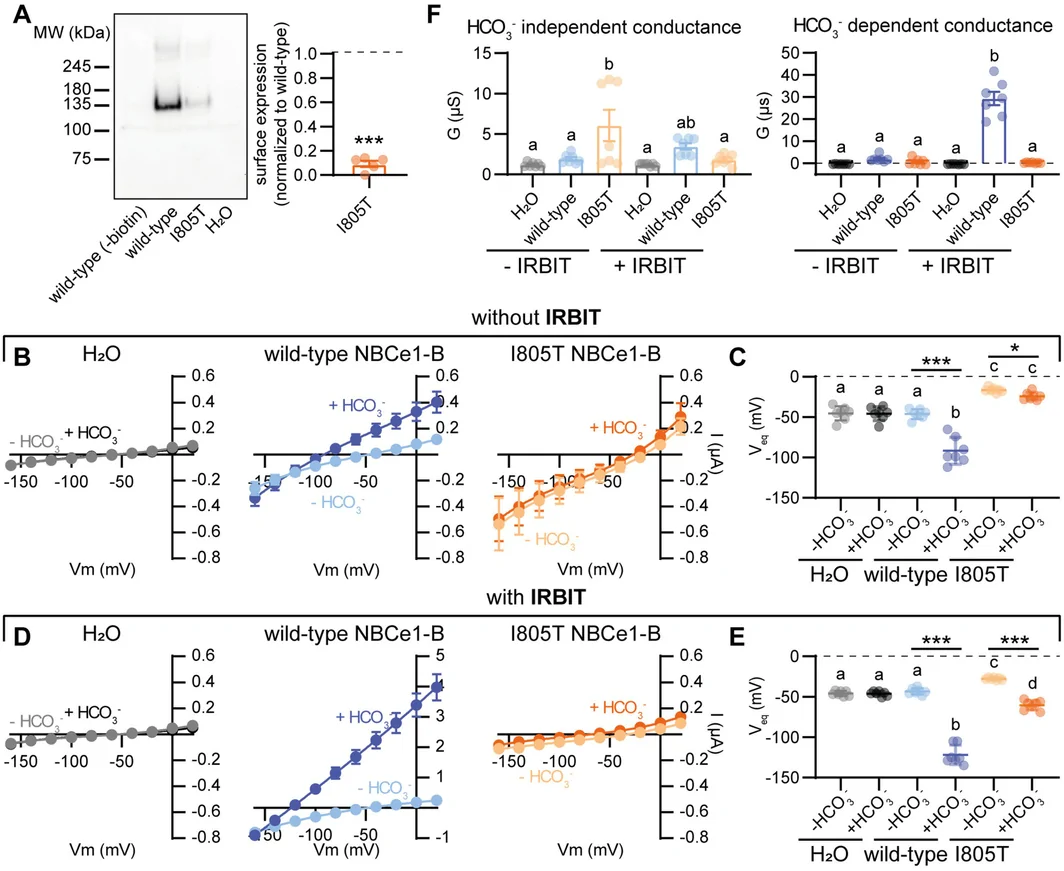
Prominent depolarizing leak and disrupted transporter function of p.(Ile805Thr) NBCe1‐B expressed in oocytes. (A) Cell surface biotinylation experiments reveal a strong reduction of p.(Ile805Thr) NBCe1‐B membrane expression in oocytes compared to wild‐type. Left: Representative western blot. Right: Summary data from multiple experiments of p.(Ile805Thr) surface expression normalized to wild‐type (0.092 ± 0.026, *n* = 5; *p* < 0.0001). (B) Averaged I/V curves measured in absence and presence of HCO_3_^−^, from oocytes injected with H_2_O (gray/black), or with cRNA encoding wild‐type (light/dark blue) or p.(Ile805Thr) NBCe1‐B (light/dark orange). (C) Summary data of I/V curve reversal potential (V_eq_). In absence of HCO_3_^−^, V_eq_ is significantly depolarized in oocytes injected with p.(Ile805Thr) NBCe1‐B when compared to H_2_O or wild‐type NBCe1‐B‐injected oocytes, showing that the variant induces a prominent depolarizing leak (V_eq_ without HCO_3_^−^: H_2_O: −45.29 ± 3.34 mV, *n* = 7; wild‐type: −46.14 ± 2.34 mV, *n* = 7; p.(Ile805Thr): −16.29 ± 1.19 mV, *n* = 7). Switching to HCO_3_^−^‐containing extracellular solution significantly hyperpolarizes V_eq_ in oocytes injected with wild‐type and, to a lesser extent, with p.(Ile805Thr) NBCe1‐B (V_eq_ with HCO_3_^−^: H_2_O: −45.71 ± 3.25 mV, *n* = 7, *p* = 0.55; wild‐type: −91.71 ± 6.32 mV, *n* = 7, *p* < 0.0001; p.(Ile805Thr): −24.00 ± 1.83 mV, *n* = 7, *p* = 0.019). (D) Same as in B, but for oocytes co‐injected with cRNA encoding the NBCe1‐B‐stimulating protein IRBIT. IRBIT does not affect HCO_3_^−^‐independent current, but greatly increases HCO_3_^−^‐dependent transporter activity for wild‐type NBCe1‐B. (E) Summary V_eq_ data for oocytes co‐injected with IRBIT. Depolarizing leak persists for p.(Ile805Thr) NBCe1‐B, albeit with reduced magnitude. The magnitude of HCO_3_^−^‐induced hyperpolarization is greatly enhanced by IRBIT, indicating stimulation of NBCe1‐B transporter activity (V_eq_ without HCO_3_^−^: H_2_O + IRBIT: −45.86 ± 1.20 mV, *n* = 7; wild‐type + IRBIT: −43.29 ± 1.51 mV, *n* = 7; p.(Ile805Thr) + IRBIT: −27.71 ± 0.57 mV, *n* = 7; V_eq_ with HCO_3_^−^: H_2_O + IRBIT: −46.14 ± 1.12 mV, *n* = 7, *p* = 0.77; wild‐type + IRBIT: −121.90 ± 4.52 mV, *n* = 7, *p* < 0.0001; p.(Ile805Thr) + IRBIT: −60.43 ± 2.21 mV, *n* = 7, *p* < 0.0001). (F) Summary data of HCO_3_^−^‐independent (left) and HCO_3_^−^‐dependent (right) conductance (G; slope of I/V curve from −20 to 20 mV). Expression of p.(Ile805Thr) NBCe1‐B significantly increases HCO_3_^−^‐independent conductance when compared to H_2_O and wild‐type NBCe1‐B conditions, indicating transporter leak (HCO_3_^−^‐independent G: H_2_O: 1.20 ± 0.10 nS, *n* = 7; wild‐type: 1.93 ± 0.22 nS, *n* = 7; p.(Ile805Thr): 6.05 ± 1.93 nS, *n* = 7). Co‐expression of IRBIT reduces HCO_3_^−^‐independent conductance in p.(Ile805Thr) NBCe1‐B, suggesting that IRBIT reduces the leak (HCO_3_^−^‐independent G: H_2_O + IRBIT: −1.20 ± 0.06 nS, *n* = 7; wild‐type + IRBIT: 3.46 ± 0.40 nS, *n* = 7; p.(Ile805Thr) + IRBIT: 1.81 ± 0.26 nS, *n* = 7). HCO_3_^−^‐dependent conductance is not detected in the absence of IRBIT in any condition (HCO_3_^−^‐dependent G: H_2_O: −0.32 ± 0.04 nS, *n* = 7; wild‐type: 1.97 ± 0.54 nS, *n* = 7; p.(Ile805Thr): 0.67 ± 0.55 nS, *n* = 7). Co‐expression of IRBIT induced a large HCO_3_^−^‐dependent conductance in wild‐type NBCe1‐B‐expressing oocytes, but not in H_2_O‐injected, nor in p.(Ile805Thr)‐injected oocytes (HCO_3_^−^‐dependent G: H_2_O + IRBIT: −0.35 ± 0.03 nS, *n* = 7; wild‐type + IRBIT: 29.32 ± 3.02 nS, *n* = 7; p.(Ile805Thr) + IRBIT: 0.24 ± 0.09 nS, *n* = 7). Dots in bar graphs and scatter plots indicate individual values. Data are presented as mean ± SEM. Asterisks indicate statistical significance (*p < 0.05, **p < 0.01, ***p < 0.001). Letters in C, E and F indicate grouping information using the Tukey method. Means that do not share a letter are significantly different.

We measured the electrophysiological properties of wild‐type and mutant NBCe1‐B in oocytes. Oocytes lack endogenous NBCe1 [24]; accordingly no surface expression of NBCe1‐B was detected in H_2_O‐injected oocytes. Comparison of oocytes injected with wild‐type or p.(Ile805Thr)‐NBCe1‐B cRNA revealed that surface expression of the mutant transporter was reduced to ~10% of that of wild‐type (Figure 6A).

Two‐electrode voltage clamp recordings in oocytes were performed to assess transporter activity and test the putative depolarizing leak observed in HEK293 cells. In the absence of HCO_3_^−^, oocytes injected with wild‐type NBCe1‐B cRNA showed similar electrophysiological properties compared to H_2_O‐injected oocytes, which lack endogenous electrogenic HCO_3_^−^ transporter activity (Figure 6B,C,F). Upon application of extracellular HCO_3_^−^, clear transporter activity was observed, which was reflected by a significant hyperpolarization of V_eq_ from ~−45 mV to ~−90 mV (Figure 6B,C). V_eq_ in the presence of HCO_3_^−^ approximated the theoretically expected value for NBCe1 (~−115 mV, Figure 6B,C) [25]. While transporter activity in NBCe1‐B‐transfected oocytes was sufficient to cause a hyperpolarization of V_eq_, it was insufficient to cause a significant change in membrane conductance (slope of the I/V curve; Figure 6F), due to the aforementioned auto‐inhibitory domain. Compared to wild‐type NBCe1‐B cRNA injected oocytes, oocytes injected with p.(IleI805Thr)‐NBCe1‐B cRNA already showed a depolarized V_eq_ and an increased basal membrane conductance in the absence of HCO_3_^−^ (Figure 6B,C,F). Both observations are in line with the putative depolarizing ion leak that we observed in HEK293 cells. Switching to HCO_3_^−^‐containing extracellular solution induced a small shift in V_eq_ and no increase in conductance for p.(IleI805Thr)‐NBCe1‐B‐containing oocytes (Figure 6B,C,F). This indicates that, in addition to the constitutive depolarizing leak, p.(IleI805Thr)‐NBCe1‐B is impaired in its HCO_3_^−^ transporter function, presumably mainly because of its greatly reduced membrane expression.

To probe the ionic nature of the depolarizing leak, we examined membrane potential deflections in H_2_O‐injected and p.(Ile805Thr)‐NBCe1‐B–expressing oocytes in response to defined extracellular ion substitutions. These experiments indicate that the leak is sufficiently large to overwhelm endogenous K^+^ permeability, shows an anion permeability comparable to the endogenous Cl^−^/gluconate permeability ratio, and displays increased Na^+^ selectivity relative to the endogenous cation permeability. Together, these findings argue against a highly selective ion channel but support a mixed, weakly selective depolarizing leak (Table S6 and accompanying text).

The IP3R‐binding protein released with inositol 1,4,5‐trisphosphate (IRBIT) interacts with NBCe1‐B and neutralizes its auto‐inhibitory domain, which greatly enhances transporter activity [26, 27]. To investigate how IRBIT influences the properties of p.(Ile805Thr)‐mutant NBCe1‐B, oocytes were co‐injected with both NBCe1‐B and IRBIT cRNA. As expected, in wild‐type NBCe1‐B‐expressing oocytes co‐expression of IRBIT greatly increased HCO_3_^−^‐dependent membrane conductance (Figure 6D–F). Co‐expression of IRBIT together with p.(Ile805Thr)‐NBCe1‐B slightly reduced the HCO_3_^−^‐independent depolarization of V_eq_ and abolished the increase, compared to H_2_O‐injected cells, in basal membrane conductance (Figure 6D–F). This suggests that IRBIT slightly reduces the depolarizing leak in the mutant transporter. HCO_3_^−^‐dependent transporter activity through the mutant transporter resulted in a modest hyperpolarization of V_eq_, but did not lead to a measurable increase in conductance.

Experiments on oocytes expressing NBCe1‐A revealed that this isoform is similarly affected as NBCe1‐B (Figure S6).

**FIGURE 7:**
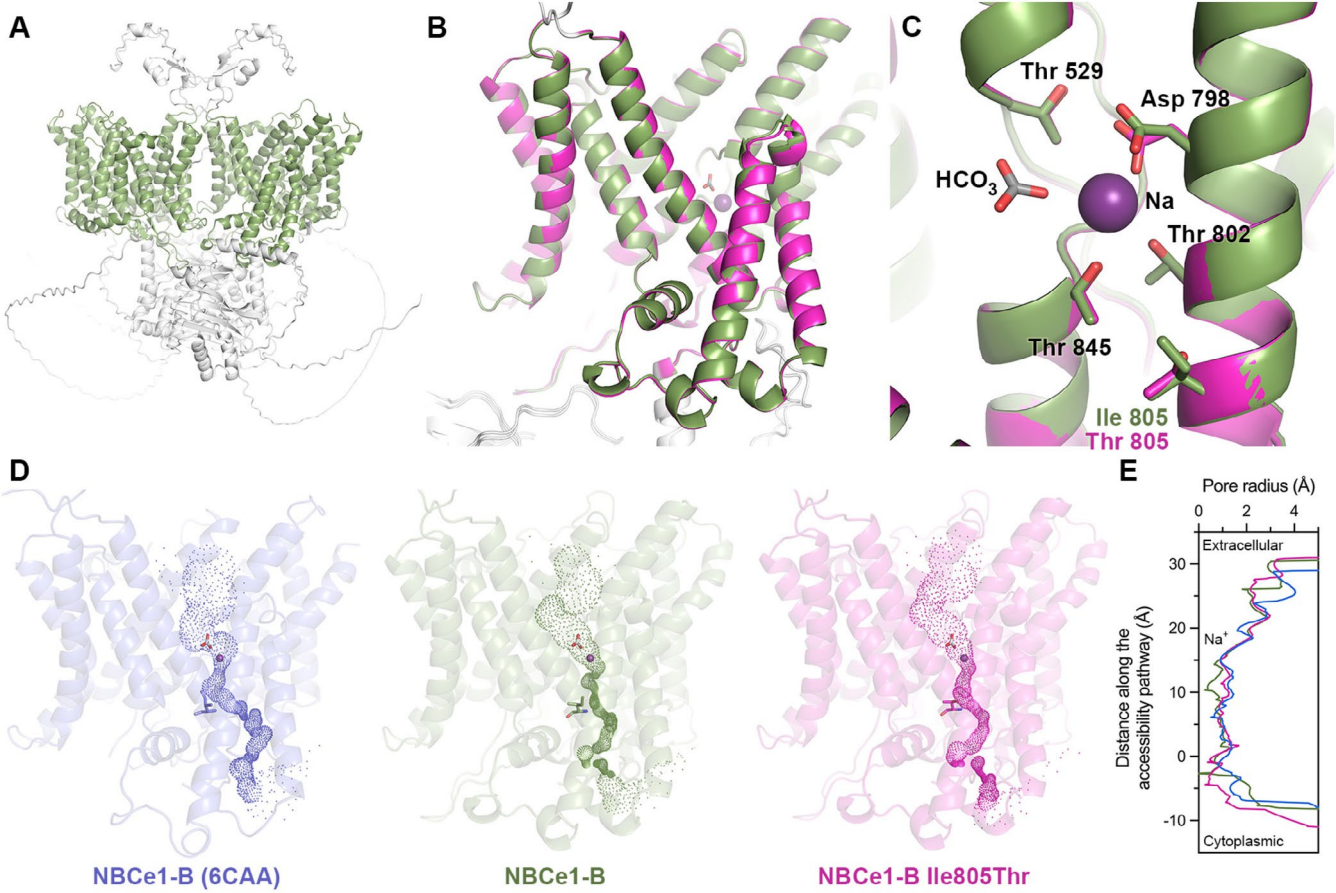
AlphaFold models of wild‐type and mutant NBCe1‐B. (A) Cartoon representation of the AlphaFold model of the wild‐type NBCe1‐B homodimer. Transmembrane domains are shown in green and cytoplasmic and extracellular domains in gray. (B) Overlay of the predicted structure of the transmembrane domains of wild‐type NBCe1‐B (green) and p.(Ile805Thr) NBCe1‐B (magenta). To indicate the location of the substrate binding pocket, HCO_3_^−^ (gray sticks) and Na^+^ (purple sphere) are modeled based on the cryo‐EM structure of the substrate‐bound sodium‐driven chloride/bicarbonate exchanger NDCBE (7RTM). (C) Zoom‐in on the substrate binding site with proposed ion coordinating residues and the patient variant shown in stick representation. (D) Comparison of the pore profile of the NBCe1 cryo‐EM structure (6CAA, blue), the AlphaFold model of wild‐type NBCe1‐B (green), and the AlphaFold model of p.(Ile805Thr) NBCe1‐B (magenta). The outline of the pore as calculated by HOLE [18] is represented by small spheres. The patient variant site is shown in stick representation. (E) Plot showing the pore radii for the structures in D. All structures show a similar pore profile for the transmembrane domain with the substrate binding site (indicated by Na^+^) being exposed to the extracellular side.

We performed structural modeling of wild‐type and p.(Ile805Thr)‐NBCe1‐B homodimers using AlphaFold 3 (Figure 7A, Figure S7) [17]. The predicted structures of the wild‐type and mutant NBCe1‐B transmembrane domains were highly similar to the cryo‐EM‐structures of NBCe1 [22] and NDCBE [28], both of which adopt an outward‐facing conformation, exposing the substrate binding pocket to the extracellular side only (Figure 7B, Figure S7). Since the substrate binding residues in NDCBE are strictly conserved in NBCe1‐B, Na^+^ and HCO_3_^−^ were modeled based on their position in the NDCBE structure, with Thr529, Thr802, Thr845 and Asp798 as putative Na^+^‐ligands (Figure 7C). The p.(Ile805Thr) variant site is located along the predicted substrate translocation pathway, immediately below the ion coordination site (Figure 7C, Figure S7), but does not seem to cause any structural alterations to this pathway. To verify this, the dimensions of the substrate‐conducting pathway were analyzed using the program HOLE [18]. As seen in Figure 7D,E, the calculated pore profiles for wild‐type and mutant NBCe1 were similar to each other and corresponded well to the experimental NBCe1‐structure, with the pore narrowing to around 1 Å on the cytoplasmic side of the substrate binding site. This is in agreement with the outward‐facing conformation, in which the substrate‐binding pocket is closed toward the cytoplasmic side. A slight difference was observed when comparing the predicted and experimental wild‐type NBCe1 structures, with the predicted structure becoming somewhat narrower around Ile805. This is interpreted as a modeling artifact due to AlphaFold choosing a different rotamer of the Ile side‐chain and not of any structural or functional relevance. In summary, the AlphaFold modeling did not predict any significant structural differences between wild‐type NBCe1‐B and the p.(Ile805Thr) variant that could lead to the appearance of a permanent ion pore or otherwise explain the depolarizing leak in the mutant protein.

## 4 Discussion

This study identifies a novel form of genetic brain edema linked to a specific bicarbonate transporter defect. We report three unrelated pediatric patients with a shared heterozygous variant in *SLC4A4*, encoding electrogenic sodium bicarbonate cotransporter NBCe1. Clinically, affected individuals present with progressive macrocephaly, episodes of raised intracranial pressure, cognitive and motor impairment, autism, and epilepsy. MRI measures show brain edema, involving cortex before white matter. Notably, two patients show clinical and radiological improvement under bicarbonate treatment. Immunohistochemistry confirms that NBCe1 is mainly expressed in astrocytes, more in cortex than in white matter. Functional studies demonstrate impaired membrane localization of mutant NBCe1, loss of bicarbonate transport activity, and a constitutive depolarizing ion leak. These drastic effects are in accordance with the amino acid substitution affecting transporter ion accessibility and coordination sites [22]. Together, these findings establish astrocyte NBCe1 as a critical molecular link between acid–base regulation and brain volume regulation.

Biallelic loss‐of‐function *SLC4A4* variants cause pRTA. Our patients are heterozygous for a dominant *SLC4A4* variant and show no clinical and only minor laboratory signs of pRTA. These findings indicate that the p.(Ile805Thr) variant exerts a pathogenic effect distinct from classic loss‐of‐function. The observed slight systemic acidosis likely results from the depolarizing leak of the mutant transporter, which is expected to reduce the driving force for renal HCO_3_^−^ reabsorption by NBCe1 encoded by the unaffected allele. The lack of non‐renal signs suggests that p.(Ile805Thr)‐NBCe1 exerts little dominant‐negative effect on wild‐type NBCe1. Consistent with this, loss‐of‐function variants in pRTA typically disrupt NBCe1 membrane localization and transporter function [29], and heterozygous carriers are unaffected. The healthy *SLC4A4* allele in our patients likely prevents non‐renal signs of pRTA.

Our patients present with infantile‐onset macrocephaly and MRI showing cerebral white matter abnormalities with swelling and anterior temporal cysts, resembling MLC. However, several features distinguish the two disorders. Most notably, medulla lesions are present in *SLC4A4*‐related encephalopathy but absent in MLC. Timing and progression of macrocephaly and edema differ as well. In MLC, macrocephaly starts within a few months after birth and is initially severe, with head circumference over +2SD before 6 months. Head growth rate becomes normal from ~12 months, with growth above but parallel to +2SD. From the onset, cerebral white matter edema is diffuse and the degree of white matter swelling explains the macrocephaly. After ~12 months, white matter swelling slowly decreases, parallel to the normalized head growth rate [30]. In *SLC4A4*‐related encephalopathy, the macrocephaly reaches +2SD later, at ~12 months, but head growth remains excessive, reaching higher SDs. The macrocephaly is initially not explained by the minor white matter abnormalities but by increased cortex volume. Only secondarily, white matter abnormalities increase, while also cortex volume continues to increase, both contributing to further increasing macrocephaly. Thus, while edema in MLC is restricted to white matter, follows the time course of myelination, and persists lifelong, edema in *SLC4A4*‐related encephalopathy involves cortex first, subsequently also white matter, has a later onset than myelination, continues to increase when myelination is largely complete, but subsides more completely later. These distinct patterns suggest different underlying mechanisms: in MLC, macrocephaly is primarily due to intramyelinic vacuoles, which appear when myelination starts [31]; in *SLC4A4*‐related encephalopathy macrocephaly is likely due to astrocyte swelling, more severely involving cortex where NBCe1 expression is higher. Myelin vacuolization is likely secondary to astrocyte swelling and lack of buffering capacity.

MLC and *SLC4A4*‐related encephalopathy also differ in clinical disease course and severity. MLC patients are initially (almost) normal, followed by a slow decline in motor function and later also cognition [32]. By contrast, *SLC4A4*‐related encephalopathy presents with early and severe motor and cognitive impairment. These differences also argue that the *SLC4A4* defect affects the brain beyond myelin vacuolization only.

Episodes of increased intracranial pressure further distinguish the two disorders. Patients with *SLC4A4*‐related encephalopathy experience episodes of increased intracranial pressure, triggered by head trauma or minor infections. MLC patients may experience prolonged decreased consciousness following mild head trauma [33, 34], but without signs of increased intracranial pressure. Interestingly, two brothers with classic pRTA also experienced episodes of increased intracranial pressure following mild head trauma, fatal in one [35]. As this report predates the identification of *SLC4A4* as the gene associated with pRTA, the specific variant in these brothers remains unknown. Collectively, these observations suggest that both the depolarizing leak in NBCe1 and loss of transporter function hamper the brain's ability to manage acute disturbances in ion and water homeostasis.

In both *SLC4A4*‐related encephalopathy and MLC, progressive cerebral atrophy occurs with age, likely reflecting accumulating neuronal damage caused by chronic ionic/pH dysregulation. It is currently unclear why the cerebral white matter MRI signal normalizes in the long run in *SLC4A4*‐related encephalopathy, but not in classic MLC.

We confirm that astrocytes are the main brain cell type expressing NBCe1, positioning *SLC4A4*‐related encephalopathy alongside MLC as a disorder in which astrocyte‐specific dysfunction causes brain edema. In addition to astrocytic expression, Na^+^‐dependent acid–base transporters are known to contribute to CSF production in the choroid plexus [36]. While altered function of these transporters could, in principle, influence CSF dynamics, CSF spaces are normal in our patients and do not explain macrocephaly in *SLC4A4*‐related encephalopathy.

NBCe1 in astrocytes is central in both intracellular and extracellular pH regulation [8, 10], and activity‐dependent astrocyte swelling [11, 12]. Astrocytes are the only body cell type in which NBCe1 may act bi‐directionally because their resting membrane potential is close to the transporter reversal potential. The majority of astrocytes likely respond to neuronal activity with outward HCO_3_^−^ transport, while a small population with depolarized resting potential responds with inward transport [10]. Neuronal activity‐induced increases in extracellular K^+^ lead to astrocyte depolarization, and the accompanying increase in inward HCO_3_^−^ transport contributes to activity‐dependent astrocyte swelling [12]. The constitutive depolarizing ion leak introduced by the p.(Ile805Thr) variant likely shifts more astrocytes toward inward HCO_3_^−^ transport, thereby promoting swelling even in the absence of neuronal activity [10, 11, 12]. This unique astrocytic vulnerability can explain differences in presentation from pRTA. It also provides a potential mechanism for therapeutic effectiveness of bicarbonate treatment. HCO_3_^−^ appears to dampen the leak in oocytes; similarly, a rise in extracellular HCO_3_^−^ could reduce the depolarizing leak in astrocytes, restore HCO_3_^−^ efflux, and reduce astrocyte swelling. Definitively establishing whether chronic astrocyte swelling underlies the observed brain edema and pinpointing the mechanism of action of bicarbonate will require studies in genetically modified animal models.

AlphaFold modeling did not resolve the structural basis for the depolarizing leak. The p.(Ile805Thr)‐variant targets a residue just below the ion coordination site, raising the possibility of subtle conformational disruption [22]. A similar pathogenic variant in *SLC4A1*, encoding red cell chloride bicarbonate anion exchanger AE1, causes red cell stomatocytosis [37]. The p.(Leu687Pro)‐variant in AE1, targeting a residue two positions downstream of the one corresponding to the mutated isoleucine in NBCe1 in our patients, converts AE1 into a non‐selective cation leak pathway [37]. In our structural models, wild‐type and mutant NBCe1 display similar pore profiles, with the substrate‐binding pocket exposed toward the extracellular side, suggesting that the leak does not arise from formation of a direct ion pore through NBCe1. The absence of a structural correlate for the leak parallels the situation for the related electroneutral sodium bicarbonate cotransporter NBCn1: the recent cryo‐EM structure with computational model for NBCn1 likewise does not reveal a structural basis for the non‐specific cation leak associated with that transporter in oocytes and HEK cells [38, 39, 40].

Transporters function by switching between outward‐ and inward‐facing conformations, thereby exposing their substrate binding site to alternating sides of the membrane [41]. Moreover, Na^+^‐driven transporters must ensure binding of both substrates (Na^+^ and HCO_3_^−^ in the case of NBCe1) before translocation. The variant may alter the conditions governing this conformational switch, resulting in incomplete coupling and an uncoupled ion leak. This hypothesis requires validation through dynamic modeling and functional experiments.

In conclusion, our study identifies a novel mechanism of brain edema driven by a depolarizing leak in NBCe1‐B, which disrupts astrocyte‐mediated regulation of pH, ion, and water homeostasis. The contrast between *SLC4A4*‐related encephalopathy and pRTA highlights that different variants in the same gene can cause organ‐specific disease phenotypes. Improvement upon bicarbonate treatment shows that the course of this new disease is modifiable. These findings underline the strong link between astrocyte acid–base regulation, ion and water homeostasis and brain edema, and uncover how a single defective astrocyte transporter can destabilize the delicate interplay between pH and brain volume.

## Author Contributions

Q.B. and S.K. designed, performed, and analyzed functional studies in HEK293 cells. M.D.P. designed, performed, and analyzed functional studies in oocytes together with R.A.P. and J.T. M.Br. performed immunohistochemistry and immunofluorescence studies on human tissue, supervised by M.Bu. G.M.v.R.‐v.L. performed western blot studies on human tissue. D.T. and E.S. studied patients 3 and 4. A.D. studied patient 2. J.A.E.v.W. supervised laboratory studies on kidney function. S.T.‐H. designed and performed AlphaFold modeling studies. P.J.W.P. designed and performed quantitative MRI studies. Q.W. designed and supervised genetic studies. M.S.v.d.K. identified the patients, identified the unique MRI pattern, studied patients 1 and 2, and supervised the MRI, genetic, and clinical studies. R.M. supervised the functional studies. M.S.v.d.K. and R.M. acquired funding, coordinated studies after gene identification, and wrote the first draft of the paper. All authors commented on the draft and approved the final version of the paper.

## Funding

This work was supported by the National Institutes of Health (EY028580) and ZonMw (40‐00812‐98‐11005, 91718392).

## Conflicts of Interest

The authors declare no conflicts of interest.

## Supporting information


**Data S1:** acn370363‐sup‐0001‐Supinfo.pdf.

## Data Availability

The data that support the findings of this study are available from the corresponding authors upon reasonable request.
