# Supplementary material for: A Depolarizing Leak in Sodium Bicarbonate Cotransporter NBCe1 Causes Brain Edema
Source: Ann Clin Transl Neurol. 2026 Mar 17:10.1002/acn3.70363. Online ahead of print. doi: 10.1002/acn3.70363 (PMC13394549; doi:10.1002/acn3.70363)
Supplement: Supplementary file 1 — Data S1: acn370363‐sup‐0001‐Supinfo.pdf. [file ACN3-9999-0-s001.pdf]

Supporting Information

A depolarizing leak in sodium bicarbonate cotransporter NBCe1 causes brain edema

Quinty Bisseling[1,2,†], Mark D. Parker[3,4,†], Sven Kerst[1,2,†], Richard A. Pasternack[3], Jacob Tondreau[3], Marjolein Breur[1], Gemma M. van Rooijen-van Leeuwen[1], Davide Tonduti[5,6], Ettore Salsano[7], Alejandra Darling[8], Joanna A.E. van Wijk[9], Susanna Törnroth-Horsefield[10], Marianna Bugiani[1,11], Petra J.W. Pouwels[12], Quinten Waisfisz[13], Marjo S. van der Knaap[1,2,*], Rogier Min[1,2,*]

†These authors contributed equally. *Senior authors.

Table of Contents

Methods S1

Study oversight page 3
Genetic analysis page 3
MRI page 3
Laboratory investigations page 4
Western Blots page 4
Immunohistochemistry and immunofluorescence page 4
Plasmids page 6
Cell lines page 6
Oocyte preparation page 6
RNA preparation and injection into oocytes page 6
Electrophysiology page 7
Cell surface biotinylation page 7
Structural modeling page 8
Statistics page 8

Figures S1-S7

Figure S1: Brain MRI in patients 3 and 4. page 9
Figure S2: Expression of *SLC4A4* in body and brain. page 10
Figure S3: Original gels belonging to Figure 4A. page 11
Figure S4: Expression of NBCe1 in the developing human brain. page 12
Figure S5: Regional expression of NBCe1 in the human brain. page 13
Figure S6: NBCe1-A is similarly affected as NBCe1-B by p.(Ile716Thr). page 14
Figure S7: Structural modeling of NBCe1-B using AlphaFold. page 15

Tables S1-S6

Table S1: Absolute volumes of brain structures. page 16
Table S2: Volumes of brain structures normalized by total intracranial volume. page 16
Table S3: Quantitative DTI measures of selected white matter structures. page 17
Table S4: Quantitative DTI measures of selected gray matter structures. page 18
Table S5: Laboratory findings for patients 1 and 2. page 19
Table S6: and accompanying text: Ion substitution analysis of the p.(Ile805Thr)-NBCe1-B depolarizing leak. page 20

Methods S1

Study oversight

We identified three patients with infantile-onset macrocephaly and the same MRI pattern of edema of the subcortical cerebral white matter and signal abnormalities in the pyramids in the medulla. With approval of the Institutional Review Board and written informed consent from the families, we reviewed their clinical and laboratory findings and performed genetic studies.

Genetic analysis

We performed trio-whole exome sequencing (trio-WES) on genomic DNA from two unrelated patients, patients 1 and 2, and their parents. Trio-WES was performed on a HiSeq 4000 or NovaSeq 6000 Sequencing System (Illumina, San Diego, CA, USA), with 150 nt paired-end sequencing. Enrichment was performed with SeqCap EZ MedExome kit or KAPA HyperExome (Roche, Basel, Switzerland), according to the manufacturer's specifications. Sequencing data was processed using an in-house bioinformatics pipeline and sequencing data were analyzed as described.[1] Variant filtering was executed under the hypothesis of autosomal recessive or autosomal dominant (*de novo*) inheritance. We focused on rare variants, exonic as well as intronic, filtering for a minor allele frequency (MAF) of less than 1% for recessive inheritance and less than 0.1% for dominant inheritance in gnomAD v2.0.2 (gnomad.broadinstitute.org). We analyzed patient 3 and his mother for the same gene variant as identified in patients 1 and 2 by Sanger sequencing.

MRI

Quantitative MRI studies were performed in patients 1 and 2. Patient 1 was scanned at a GE 3T scanner (MR750, General Electric, Milwaukee, WI, USA) at ages 1y8m, 2y2m, 2y7m, and 3y8m, and at a Philips 3T scanner (Ingenia Elition X, Philips Medical Systems, Best, the Netherlands) at ages 5y7m, 6y5m, and 7y. Patient 2 was scanned at a GE 1.5T scanner (Signa explorer) at ages 12m, 1y7m, and 2y5m, and at Philips 3T at 3y8m, 4y3m, and 4y9m. The acquisition protocol included structural MRI (3D T1-weighted images and 2D or 3D FLAIR) and at 3T also diffusion-weighted imaging (DWI) (GE protocol: single-shell DWI with b-values 0, 750 s/mm^2;[2] Philips protocol: multi-shell DWI with b-values 0, 1000, 2000 s/mm^2).[3]

Normal age-matched values were determined from 16 MRI exams at Philips 3T of young patients between 2y and 6y7m, with normal MRI and normal neurological evaluation.[3]

Analysis was performed as outlined in Stellingwerff *et al* (2024).[3] Volumes of cerebral white matter, cerebral cortex, deep gray matter, cerebellum, and CSF were determined with Synthseg,[4] using 3D T1 weighted images. In some exams with severe T1-hypointensities, segmentation of 3D T1 was not successful, leaving parts of the brain unsegmented (patient 1, between age 2y7m and 6y5m). In these cases Synthseg was more reliable using 3D FLAIR (3D FLAIR was not available at age 3y8m and this exam was not included for quantitative analyses).

Quantitative maps of fractional anisotropy (FA), mean, axial and radial diffusivity (MD, AD, and RD) were calculated with tools from FMRIB's Software Library (FSL version 6.0.4, Oxford, UK).[5, 6] In the case of multi-shell DWI this analysis was based on the single b0-b1000 shell.

Regions-of-interest (ROIs) defined on structural 3D T1-weighted MRI were registered to diffusion images, using Advanced Normalization Tools (ANTs, version 2.3.5, Pennsylvania, Philadelphia, USA).[7]

ROIs of corpus callosum were based on registration with the JHU atlas. To reduce partial volume effects of CSF or white matter, ROIs of cortex and deep gray matter structures were first eroded in 3D T1, and subsequently registered to diffusion images. Median values of quantitative measures were determined per ROI.

Laboratory investigations

Because of the genetic findings, laboratory investigations focused on renal tubular acidosis were performed in patients 1 and 2.

Western Blots

To study expression of NBCe1 protein level in healthy individuals, tissue derived from autopsy was used. For both control 1 (25-year-old male, sickle cell crisis) and control 2 (25-year-old male, drowning) tissue from frontal gray matter and white matter was used. From control 3 (21-year-old male, caffeine intoxication) kidney was used. Tissue samples were homogenized using a lysis buffer with 2 mM dithiothreitol, 1% Halt protease and phosphatase inhibitor cocktail (Fisher Scientific), 5 mM EDTA, 1% IGEPAL and 1% sodium deoxycholate and centrifuged for 10 minutes at 4°C to remove nuclei, membranes and debris. Total protein in samples was determined by Bradford assay. 25 µg of total protein was loaded into SDS-PAGE and transferred onto polyvinylideenfluoride (PVDF) membranes. Protein loading was checked with 2,2,2-trichloroethanol (TCE). Membranes were blocked with 5% non-fat-dry-milk and incubated overnight (ON) at 4°C with primary antibodies NBCe1-A (1:500, kind gift from Dr. Michael Romero, Mayo Clinic College of Medicine & Science, Rochester, USA), NBCe1-B/C (1:1000, SC-515543, Santa Cruz) and NBCe1-pan (1:1000, E-AB-14348, Elabscience). Horseradish peroxidase (HRP)-linked secondary antibodies were used to develop the immunoblots and the signal was detected with an enhanced chemiluminescent substrate (SuperSignal West Femto Substrate, Fisher Scientific). Images were taken with the Bio-Rad ChemiDoc imaging system.

Immunohistochemistry and immunofluorescence

To study expression of NBCe1 in the human brain, tissue samples from the frontal lobe were obtained at autopsy from three control patients; a 7-month-old female patient (status epilepticus), a 28-year-old male (sinus thrombosis) and a 43-year-old male (myocardial infarction). To guarantee antibody specificity a kidney sample was used from a 21-year-old male patient who died of caffeine intoxication.

To investigate NBCe1 expression in the developing human brain, frontal lobe tissue samples were collected from patients at various stages of development. These included fetal brain tissue at 23 and 40 weeks of gestation, both affected by chorioamnionitis. Additionally, samples were obtained from a 3-year-old female with meningitis, a 5-year-old female with encephalitis, an 8-year-old male with myocardial infarction and edema, and a 12-year-old male with myocarditis and edema.

None of the control subjects had neurological symptoms. Routine neuropathological examination (gross examination of brain anatomy as well as review of Hematoxylin and Eosin-stained tissue sections from multiple brain areas) was normal.

For immunohistochemistry on human tissue, formalin-fixed paraffin-embedded (FFPE) frontal lobe tissue of control patients was cut in 5 µm-thick sections. Immunohistochemical staining was carried out by de-paraffinizing sections in xylene and rehydrated in descending concentrations of ethanol. Endogenous peroxidase activity was blocked in 0.3% (w/v) H_20_2 in PBS for 30 minutes, followed by heat-induced antigen retrieval in TRIS/EDTA buffer (pH = 9) using an autoclave. Primary antibodies

against NBCe1-A (1:100, kind gift from Dr. Michael Romero, Mayo Clinic College of Medicine & Science, Rochester, USA), NBCe1-B/C (1:100, SC-515543, Santa Cruz) and NBCe1-pan (1:50, E-AB-14348, Elabscience) were incubated ON at room temperature (RT). The following day, slides were rinsed and incubated with horseradish peroxidase labelled secondary antibodies and developed using 3,3′-diaminobenzidine (DAB, 1:50, Agilent) for 10 minutes. Sections were counterstained with haematoxylin, dehydrated with ethanol and xylene and mounted using PERTEX (KAM-0801, VWR). Light microscopy images were taken using an Olympus RX50 microscope (Evident Europe GmbH, Leiderdorp, The Netherlands).

Immunofluorescent staining of FFPE tissue sections was carried out by de-paraffinizing sections in xylene followed by rehydration through a graded ethanol series. Crosslinking of formaldehyde was quenched by immersing slides in 0.1% glycine solution for 10 minutes at RT, followed by heat-induced antigen retrieval in TRIS/EDTA buffer (pH = 9) using an autoclave. Sections were stained ON at RT with NBCe-1 (1:50, E-AB-14348, Elabscience) combined with various cell markers: glial fibrillary acidic protein (GFAP) for astrocytes (1:500, AB4674, Abcam), microtubule-associated protein 2 (MAP2) for neuronal cells and dendritic processes (1:200, 13-1500, Invitrogen), cluster of differentiation 68 (CD68, KP1 clone) for cells of monocyte lineage and macrophages, (1:500, M0814, Agilent) and Ulex europaeus Agglutinin I (ULEX) as a marker for vascular endothelium (1:100, B-1065-2, Vector laboratories). After PBS washes, sections were incubated with corresponding fluorescent secondary antibodies for 1 hour at RT. To minimize autofluorescence, sections were incubated with Sudan black (0.1% in 70% EtOH) for 10 minutes, then counterstained and mounted with DAPI Fluoromount-G (0100-20, Southern Biotech). Fluorescent images were photographed using a Leica DM5000B microscope (Leica Microsystems BV, Rijswijk, The Netherlands).

For immunofluorescent staining of HEK293 cells, 48 hours after transfection cell membranes were stained before fixation using a wheat germ agglutinin (WGA) Conjugate (WGA633, 29024-1, Biotium, USA, 2 mg/mL) dissolved at 5 µg/mL in phenol red-free Hanks's Balanced Salt Solution without calcium and magnesium ($HBSS^{-/-}$; Gibco, 14175-053) for 10 minutes in a humidified CO_2 incubator (37°C / 5% CO_2). Immediately after coverslips were washed two times with $HBSS^{-/-}$; fixated with 2% paraformaldehyde (PFA; Electron Microscopy Sciences, E15710-S) for 15 minutes at RT and washed three times with Dulbecco's Phosphate Buffered Saline (DPBS; Gibco, 14190-094), and with the third wash stained for 4′,6-diamidino-2-phenylindole (DAPI; 1:2000; Sigma, D9542, 5 mg/ml) diluted in DPBS. Coverslips were washed once with DPBS to remove excess DAPI and mounted on glass slides using ProLong^{TM} Glass Antifade Mountant mounting medium (Invitrogen, P36984), then, in the dark, stored first at RT for 24 hours and then at 4°C until use.

Analysis of NBCe1-B expression on membrane vs cytosol was determined as follows: multiple high-resolution z-stacks were obtained from stained wild-type or p.(Ile805Thr)-NBCe1-B-eGFP expressing HEK293 cells with a z step size of 0.25 µm on an inverted Nikon Eclipse T*i*2 confocal microscope (Nikon, Japan) with an oil-immersion objective (Nikon Plan Fluor 40X/NA 1.3/ WD 240 µm). DAPI was excited at 406 nm (emission filter: 425-475), NBCe1-B-eGFP at 488 nm (emission filter: 500-550) and WGA at 635 nm (emission filter: 663-738). Fluorescence quantification was performed by manually drawing lines in one z-stack plane through 20-30 transfected cells per condition using ImageJ software.[8] Care was taken to draw lines perpendicular to the membrane on both sides, without crossing the cell nucleus (for examples see Figure 5B). Fluorescence intensity for both the green (NBCe1-B-eGFP) and magenta (WGA633 membrane dye) channels was extracted. To determine membrane fluorescence, the two peaks in the magenta channel indicating the plasma membrane crossings were identified for

each line, and mean fluorescence intensity (MFI) of the green channel was determined in a 500 nm area centered on each peak. For cytosolic fluorescence, the MFI of the green channel in the central area 1 µm from each peak (defined in the magenta channel) was determined. Both membrane and cytosolic fluorescence were corrected for background fluorescence by subtracting MFI of the green channel from untransfected HEK293 cells. To calculate membrane enrichment, mean membrane fluorescence was divided by mean cytosolic fluorescence.

Plasmids

The plasmid encoding the human *SLC4A4* isoform NBCe1-B with a C-terminal eGFP tag in the pcDNA3.1 backbone was a kind gift from the lab of Dr. George Seki (Yaizu City Hospital, Japan), Dr. Shoko Horita (Tokyo University, Japan) and Dr. Osamu Yamazaki (Teikyo University, Japan).[9] The Ile805Thr variant was introduced using the Seamless Ligation Cloning Extract (SLiCE) cloning method.[10] The plasmid encoding the human *SLC4A4* isoform NBCe1-B with a C-terminal EGFP-tag in the pGH19 *Xenopus* expression vector was a kind gift from the lab of Dr. Walter Boron (Case Western Reserve University, USA).[11] The Ile805Thr variant as introduced using a QuikChange XL site-directed mutagenesis kit (Agilent Technologies Inc.).

Cell lines

HEK293 cells were cultured in Dulbecco's Modified Eagle Medium (DMEM; Gibco, 41966-029), high glucose, pyruvate, supplemented with 10% Fetal Bovine Serum (FBS; Gibco, 10270-106) and 1% Penicillin-Streptomycin (Pen/Strep; Gibco, 15140122) in a humidified CO_2 incubator (37°C / 5% CO_2), and passaged 1:10 dilution two times per week. For immunocytochemistry, cells were seeded on poly-L-lysine (PLL)-coated (0.1 mg/mL for 1-2 hours at 37°C; Sigma, P2636) glass coverslips (13 mm, #1, VWR, 631-1578) with a density of 100.000 cells per mL, for electrophysiology experiments on uncoated glass coverslips with a density of 10.000 cells per mL. Cells were transfected 24 hours after plating with wild-type or p.Ile805Thr *SLC4A4* isoform B using FuGENE HD (Promega, E2311) according to manufacturer's instructions (DNA:FuGENE ratio 1:3), mock conditions following the same transfection protocol but without adding DNA.

Oocyte preparation

The method used for harvesting of ovaries from *Xenopus laevis* frogs was approved by the Institutional Animal Care and Use Committee at the University at Buffalo. In summary, frogs were anesthetized in 0.2 % tricaine methanesulfonate solution until unresponsive to toe pinch and ovarian tissue was surgically extracted. Frogs were subsequently euthanized by cardiac excision. Ovarian tissue was cut into ~5 mm^3 pieces and washed for 3 x 5 minutes in calcium-free NRS solution (82 mM NaCl, 2 mM KCl, 20 mM MgCl2, 5 mM HEPES, pH 7.45) before a 20 minutes incubation on a rotating wheel in the same solution containing 2 mg/mL of type-1A collagenase (C2674, Sigma Aldrich). Following a further 3 x 10 minutes washes in calcium-free NRS solution, liberated oocytes are rinsed in ND96 solution (96 mM NaCl, 2 mM KCl, 1 mM $MgCl_2$, 1.8 mM $CaCl_2$, 5mM HEPES, pH 7.50). For long term culture, oocytes are maintained at 18°C in OR3 medium (14 g/L Leibovitz's L-15 medium supplemented with 5 mM HEPES, 100 units/mL penicillin, 100 µg/mL streptomycin: pH adjusted to 7.50, osmolarity adjusted to 195 ± 5 mOsmol/kg H_2O).

RNA preparation and injection into oocytes

pGH19 constructs were linearized using *NotI*, purified using MinElute PCR purification columns (QIAGEN), and used as template to produce cRNA using a mMESSAGE mMACHINE T7 transcription kit (Thermo Fisher Scientific). Each oocyte was injected with 25 ng of cRNA using a Nanoject III programmable nanoliter injector (Drummond Scientific Company). Oocytes were assayed electrophysiologically 3-5 days after injection.

Electrophysiology

Whole cell patch-clamp recordings were performed in HEK293 cells at RT, 24 hours - 48 hours after transfection. Coverslips with cells were placed in bath solution containing in mM: 110 NaCl, 10 CsCl, 1 $MgCl_2$, 1.5 $CaCl_2$, 10 HEPES, 15 glucose, 25 Na-gluconate (pH adjusted to 7.40 with NaOH and osmolality adjusted to 305 mOsm/kg). Whole cell patch-clamp recordings were made using borosilicate glass micropipettes (pipette resistance 2.5 - 4.5 MΩ) filled with an internal solution containing in mM: 125 Cs-gluconate, 10 Na-gluconate, 1 $CaCl_2$, 10 TEA-Cl, 10 HEPES, 10 EGTA, 1 Mg-ATP, 1 Na_2-ATP (pH adjusted to 7.4 with CsOH and osmolality adjusted to 295 mOsm/kg). Cells were kept at a holding potential of -60 mV, and three minutes after achieving whole cell configuration currents were measured by applying voltage steps from -100 to +60 mV (averaged over three runs, Δ_{step} = 10 mV, 30 s start-to-start interval, 600 ms step duration) with an AXOPATCH 200B amplifier and pCLAMP 10.2 software (Molecular Devices, USA). In all recordings series resistance remained <20 MΩ. I/V relationship was analyzed using GraphPad Prism 9 (GraphPad, USA). The equilibrium potential (V_{eq}) of the I/V curve was determined by fitting a third order polynomial nonlinear regression model to the I/V curve and defining the voltage at I = 0 of the fitted curve. The conductance density was determined by fitting a linear regression model to the I/V curve between +20 and +60 mV and defining the slope of the line normalized to whole cell capacitance.

Two-electrode voltage clamp data were obtained from oocytes using an OC-725C oocyte clamp under the control of pClamp 10.4 software. Oocytes were superfused with either a bicarbonate-free ND96 solution or the bicarbonate containing equivalent (in which 33 mM NaCl is replaced by 33 mM $NaHCO_3$ and adjusted to pH 7.50 by bubbling with 5% CO_2, balanced air.) Electrodes were fabricated from borosilicate glass (#BF200-156-10, Sutter Instrument) using a P-1000 micropipette puller (Sutter Instrument) such that the tip resistance was 0.2 - 2.0 MΩ when filled with 3 M KCl. Cells were clamped at spontaneous membrane potential and voltage was stepped from -160 mV to +20 mV for 100 ms, returning to spontaneous potential for 100 ms between steps.

Cell surface biotinylation

Surface expression of NBCe1-B in oocytes was determined using a Pierce Cell Surface Biotinylation and Isolation Kit (Thermo Fisher Scientific) to isolate biotinylated protein from batches of 15 oocytes. The procedure was modified for oocytes by [1] lowering the osmolarity of the PBS buffer to 200 mOsmol/kg and [2] homogenizing cells in oocyte homogenization buffer (TBS containing 1% Triton X-100 and EDTA-free protease inhibitor cocktail tablet (Millipore Sigma)) using a plastic micropestle. Protein was resolved on NuPAGE 3 - 8% Tris-Acetate gels, transferred onto PVDF membranes and western blotted using an anti-NBCe1 antibody raised in rabbits (1:1000 dilution, #E-AB-14348; Elabscience Biotechnology, Houston, TX) and a HRP-conjugated goat-anti-rabbit secondary antibody (1:1000 dilution, #55685; MP Biomedicals, Solon, OH). Antibodies were validated in an earlier study.[12] HRP signal was visualized using ECL2 western blotting substrate (Thermo Fisher Scientific) and a My ECL

Imager (Thermo Fisher Scientific) digital gel documentation system. Bands were quantified using Fiji software.[8]

Structural modeling using AlphaFold 3

Two copies of the amino acid sequence of NBCe1-B and p.(Ile805Thr)-NBCe1-B were used as input for AlphaFold 3[13] to obtain the predicted structures of the respective homodimers. The confidence of predicted structures were evaluated based on the predicted Local Distance Difference Test (pLDDT), a per-residue measure of local confidence, and the Predicted Align Error (PAE) which assesses the confidence in the relative position of amino acids within the predicted structure (Figure S7A and B). For comparison with the NBCe1-B (6CAA) and NDCBE (7RTM) experimental structures, the gate domain (residues 582-610, 692-715, 721-747, 902-918, 947-969, 971-993 in NBCe1-B) was used for alignment (Figure S7C). Na^+ and HCO_3^- were modeled inside the substrate-binding pocket based on their position in the NDCBE structure. Calculations of the pore dimensions was done using the program HOLE with xplor.rad as the radius definition file.[14] All structure figures were generated using PyMOL (The PyMOL Molecular Graphics System, Version 3.0 Schrödinger LLC, New York City, New York, USA).

Statistics

Statistical analysis on HEK293 cell data was performed using GraphPad Prism 9 (GraphPad, USA). Data were tested for normality with a Kolmogorov-Smirnov test. Normally distributed data were compared using an unpaired *t*-test, while not-normally distributed data was tested using a Mann-Whitney test. Statistical analysis on oocyte data was performed using Minitab. The General Linear Model feature was used for comparing multiple groups, followed by Tukey's method for group comparisons. Statistically significant differences were defined as $p \leq 0.05$. Data are represented as mean ± SEM.

Figures S1-S7

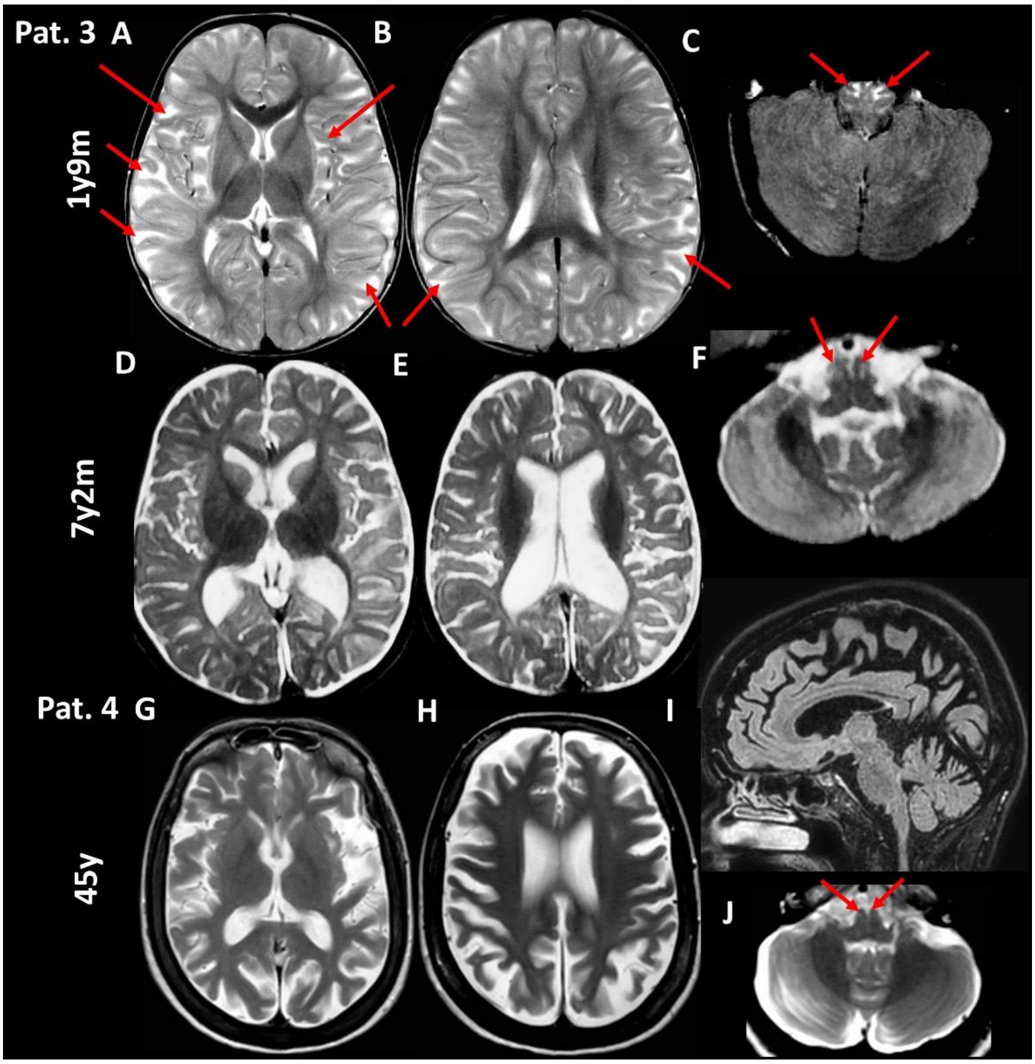

Figure S1: Brain MRI in patients 3 and 4.
(A-C) The MRI in patient 3 at 1y9m shows signal abnormalities and swelling of the subcortical and deep cerebral white matter (red arrows in A, B) and signal abnormalities in the pyramids and hilum of the inferior olives in the medulla (red arrows in C). (D-F) Follow-up MRI at 7y2m shows a decrease in cerebral white matter signal abnormalities and swelling, and atrophy associated with widening of the lateral ventricles and subarachnoid spaces (D, E). Medulla abnormalities are still present (red arrows in F). (G-J) MRI in patient 4 at 45y shows no signal abnormalities in the cerebral white matter (G, H) and medulla (red arrows in J); there is marked cerebral and cerebellar atrophy (G-J). y, years; m, months.

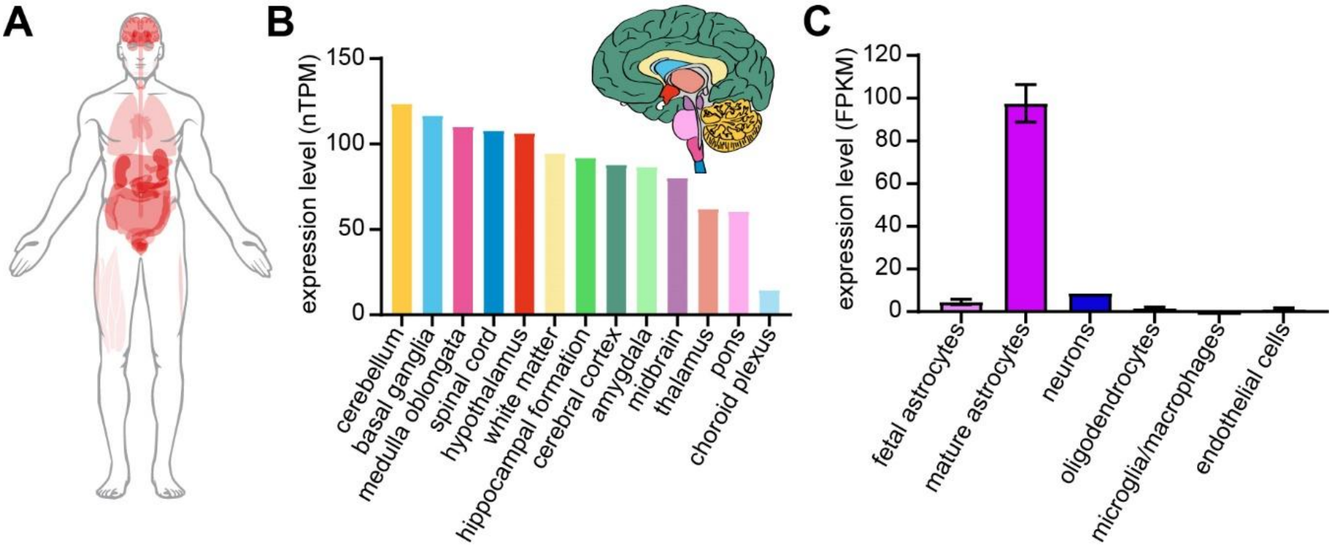

Figure S2: Expression of *SLC4A4* in body and brain.

(A) Anatogram illustrating relative expression levels of *SLC4A4* in the body. Darker red colors signify higher expression levels. (B) RNA expression levels in different areas of human brain (color coded, inset illustrates color coding). Normalized expression levels calculated as normalized total transcripts per million (nTPM). (C) *SLC4A4* expression in different cell types of the human brain. Values are depicted as RNA Fragments Per Kilobase Million (FPKM). *SLC4A4* is prominently expressed in mature astrocytes. Data and illustrations in A and B from the Human Protein Atlas (v.24.0): https://www.proteinatlas.org/ENSG00000080493-SLC4A4.[15, 16] Data in C from https://brainrnaseq.org.[17]

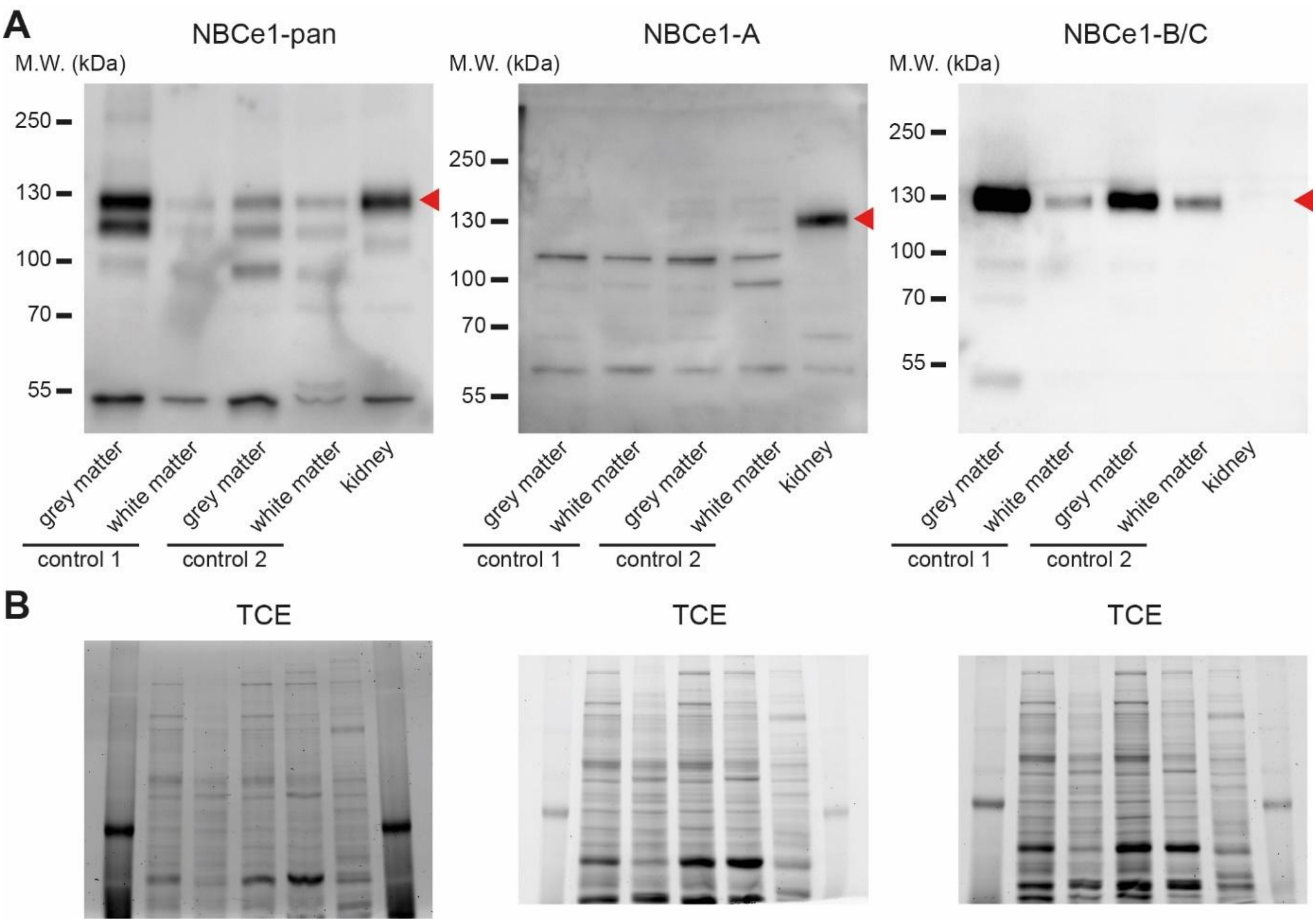

Figure S3: Original gels belonging to Figure 4A.

(A) Full western blots from human gray and white matter lysates from two different subjects and a kidney lysate from a third subject, using either an antibody recognizing all NBCe1 isoforms (NBCe1-pan; left) or only the NBCe1-A (middle) or NBCe1-B/C (right) isoforms. Red arrow indicates the expected band for NBCe1 protein (~130 kDa). (B) 2,2,2-Trichloroethanol (TCE) visualization from the gels used for A.

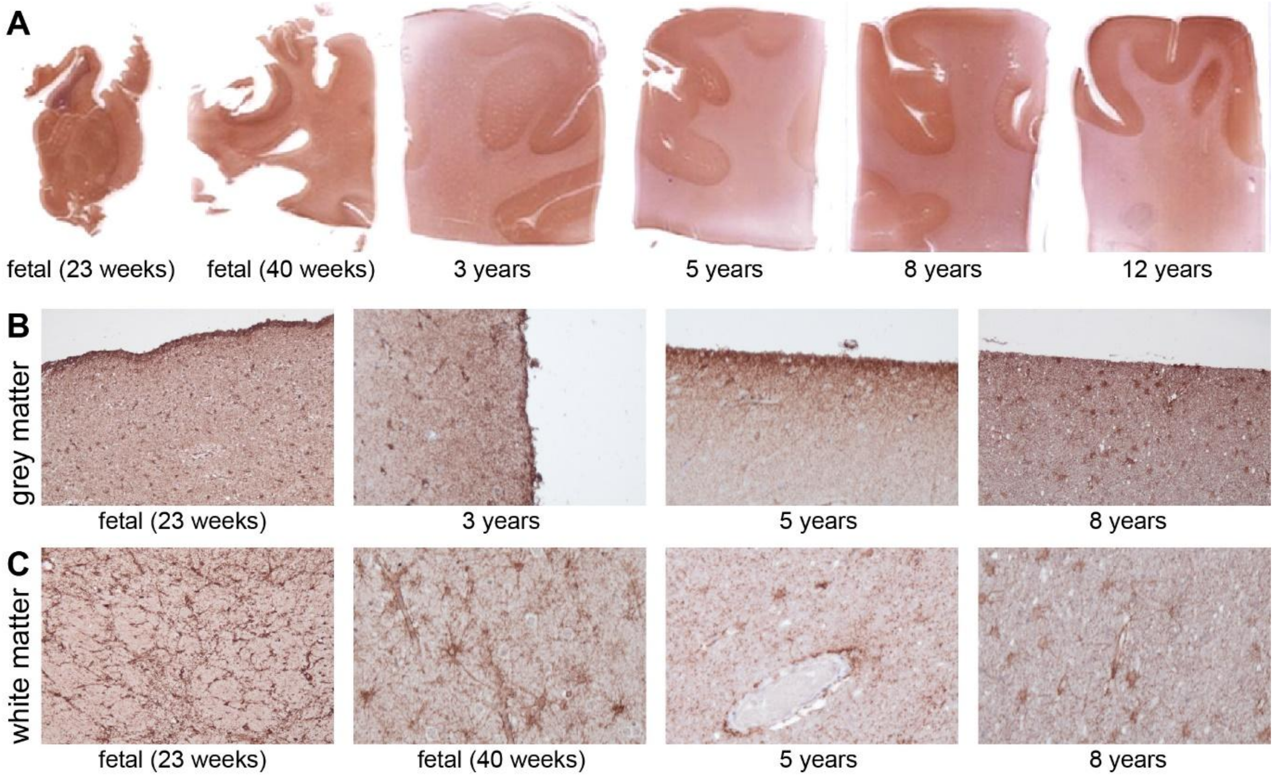

Figure S4: Expression of NBCe1 in the developing human brain.

(A) Overview images of human brain sections containing both white and gray matter, stained using the pan-isoform antibody for NBCe1. Expression is detected at all studied ages throughout the brain, with levels appearing higher in gray than in white matter. (B, C) Zoomed-in microscopy images from cortical gray (B) and white (C) matter. Stainings suggest a widespread astrocytic expression pattern, with enrichment in perivascular and subpial glia limitans.

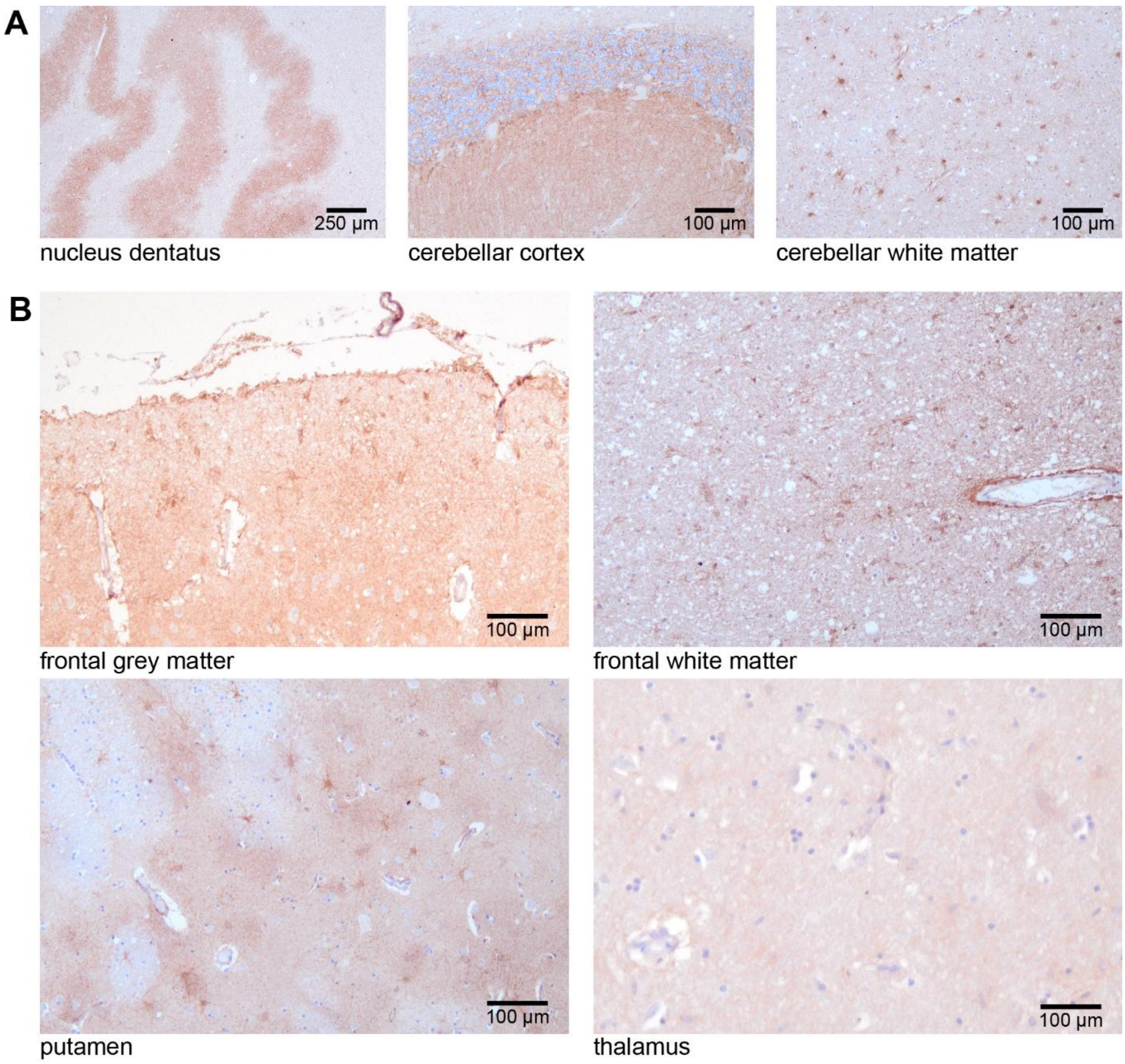

Figure S5: Regional expression of NBCe1 in the human brain.
(A) Microscopy images from human brain sections containing different regions of the cerebellum (nucleus dentatus, cerebellar cortex, cerebellar white matter), stained using the pan-isoform antibody for NBCe1. Clear DAB staining is observed throughout the nucleus dentatus, in Bergman glia and in astrocytes in the cerebellar white matter. (B) Human brain sections from the same subject, showing frontal gray matter, frontal white matter, putamen and thalamus. While it is hard to compare staining intensity and within and between subject variability is high, NBCe1 staining intensity in thalamus was lower compared to these other regions in two out of three subjects.

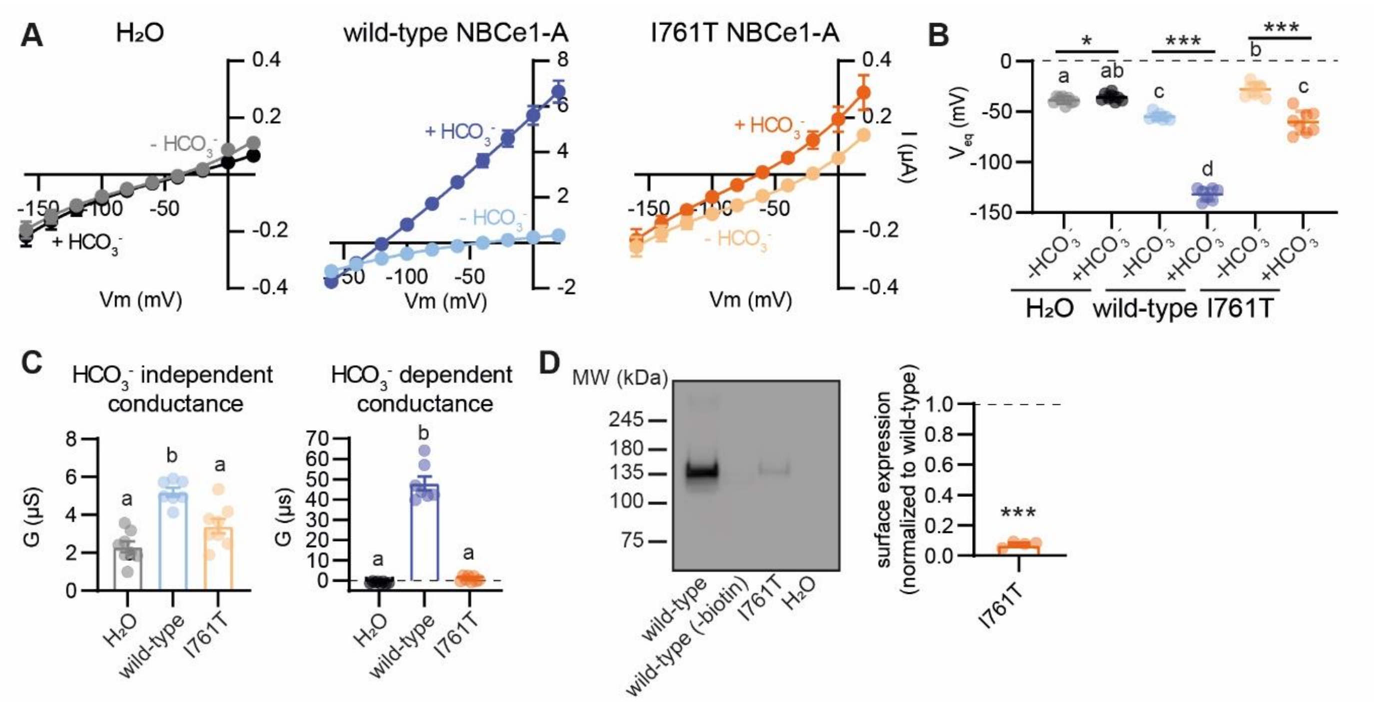

Figure S6: NBCe1-A is similarly affected as NBCe1-B by p.(Ile716Thr).

(A) Averaged I/V curves measured in absence and presence of HCO_3^-, from oocytes injected with H_2O (left: gray/black), or with cRNA encoding wild-type (middle, light/dark blue) or p.(Ile761Thr) NBCe1-A (right; light/dark orange). (B) Summary data of I/V curve reversal potential (V_{eq}). In absence of HCO_3^-, V_{eq} is significantly depolarized in oocytes injected with p.(Ile761Thr) NBCe1-A when compared to H_2O or wild-type NBCe1-B-injected oocytes. This suggests that the variant induces a prominent depolarizing leak (V_{eq} without HCO_3^-: H_2O: -38.63 ± 1.30 mV, n = 8; wild-type: -54.86 ± 1.39 mV, n = 7; p.(Ile761Thr): -28.00 ± 2.27 mV, n = 8). Switching to HCO_3^--containing extracellular solution leads to a slight but (significant) depolarization in H_2O-injected oocytes, and significantly hyperpolarizes V_{eq} in oocytes injected with wild-type and, to a lesser extent, with p.(Ile761Thr) NBCe1-A (V_{eq} with HCO_3^-: H_2O: -35.75 ± 1.39 mV, n = 8, p = 0.025; wild-type: -131.90 ± 2.30 mV, n = 7, p < 0.0001; p.(Ile761Thr): -60.50 ± 3.92 mV, n = 8, p = 0.0004). (C) Summary data of conductance (G; slope of I/V curve from -20 to 20 mV) in the absence (left) and presence (right) of HCO_3^-. Expression of wild-type NBCe1-A leads to a significant increase in both HCO_3^--independent (left) and HCO_3^--dependent (right) conductance when compared to H_2O or p.(Ile761Thr) NBCe1-A conditions (G without HCO_3^-: H_2O: 2.31 ± 0.29 nS, n = 8; wild-type: 5.19 ± 0.24 nS, n = 7; p.(Ile761Thr): 3.41 ± 1.08 nS, n = 8; G with HCO_3^-: H_2O: -0.96 ± 0.17 nS, n = 8; wild-type: 47.98 ± 3.44 nS, n = 7; p.(Ile761Thr): 0.80 ± 0.48 nS, n = 8). (D) Cell surface biotinylation experiments reveal a strong reduction of p.(Ile761Thr) NBCe1-A membrane expression in oocytes when compared to wild-type. Left: Representative western blot. Right: Summary data from multiple experiments of p.(Ile761Thr) surface expression normalized to wild-type (0.076 ± 0.009, n = 4 wild-type and 4 p.(Ile761Thr); p < 0.0001). Dots in bar graphs and scatter plots indicate individual values. Data are presented as mean ± SEM. Asterisks indicate statistical significance (* p < 0.05, ** p < 0.01, *** p < 0.001) Letters in B and C indicate grouping information using the Tukey method. Means that do not share a letter are significantly different.

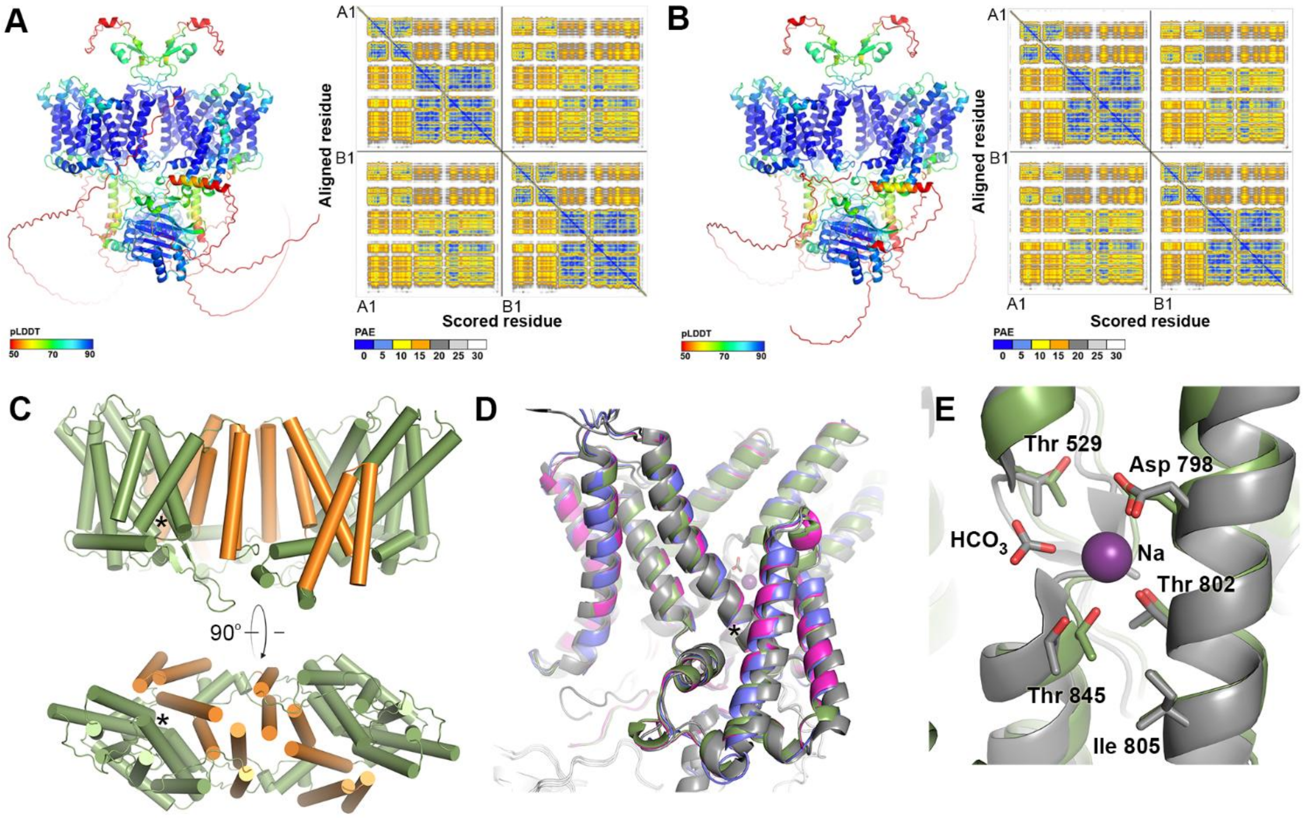

Figure S7: Structural modeling of NBCe1-B using AlphaFold.
(A,B) Predicted structures and confidence metrics of wild-type NBCe1-B (A) and (B) p.(Ile805Thr) NBCe1-B. The cartoon representations are colored according to the per-atom confidence estimate pLDDT. The diagrams on the right show the predicted aligned error (PAE) which is the estimated error in the relative position and orientation of two residues in the predicted structures. (C) Cylindrical representation of the transmembrane region of the predicted structure of wild-type NBCe1-B. Transmembrane helices 5-7 and 13-14 that constitute the gate domain and were used for structural alignment are colored orange. The approximate location of Ile805 is indicated by a black asterisk. (D) Structural overlay of transmembrane domains for wild-type NCBe1-B (AlphaFold model, green), p.(Ile805Thr) NCBe1-B (AlphaFold model, magenta), NCBe1-B cryo-EM structure (blue, 6CAA) and NDCBE cryo-EM structure (gray, 7RTM). The approximate location of Ile805 is indicated by a black asterisk. (E) Structural overlay of the substrate binding site in the predicted structure of wild-type NCBe1-B (green) and the cryo-EM structure of NDCBE (gray) showing the strict conservation of residues involved in ion coordination and the patient variant p.(Ile805Thr). CO_3^{2-} (CO_3) and Na^+ from the NDCBE structure are shown in stick and sphere representation, respectively.

Tables S1-S6

Table S1: Absolute volumes of brain structures.

Subjects	Patient 1							Patient 2							Controls
Age	1y8m	2y2m	2y7m	5y7m	6y5m	7y	8y	12m	1y7m	2y5m	3y8m	4y3m	4y9m	5y9m	3y7m (14m)
total intracranial	1633	1839	2029	2360	2362	2379	2229	1443	1757	2153	2427	2453	2452	2328	1468 (114)
cerebral white matter	379	438	559	659	639	626	563	365	455	694	785	794	768	611	399 (40)
cerebral cortex	701	768	846	999	965	950	849	579	697	802	912	907	905	790	550 (40)
deep gray matter	44	46	43	47	49	48	50	41	41	49	53	51	52	53	43 (3)
cerebellum	136	167	161	155	154	151	144	124	131	145	124	125	124	121	141 (11)
CSF	327	362	372	433	475	511	504	286	369	392	481	495	512	608	289 (27)

Volumes are in ml. The control values are indicated as mean (SD). An age in red indicates that the patient was on treatment for at least 3 months. Yellow indicates values larger than 2 SD (light colors) or 3 SD (dark colors) of the control range. y, years; m, months.

Table S2: Volumes of brain structures normalized by total intracranial volume.

Subjects	Patient 1							Patient 2							Controls
Age	1y8m	2y2m	2y7m	5y7m	6y5m	7y	8y	12m	1y7m	2y5m	3y8m	4y3m	4y9m	5y9m	3y7m (14m)
cerebral white matter	23.2	23.8	27.6	27.9	27.0	26.3	27.6	25.3	25.9	32.3	32.4	32.4	31.3	26.3	27.2 (1.2)
cerebral cortex	42.9	41.8	41.7	42.3	40.8	39.9	41.7	40.1	39.7	37.2	37.6	37.0	36.9	33.9	37.5 (0.9)
deep gray matter	2.7	2.5	2.1	2.0	2.1	2.0	2.1	2.9	2.3	2.3	2.2	2.1	2.1	2.3	2.9 (0.1
cerebellum	8.3	9.1	7.9	6.6	6.5	6.4	7.9	8.6	7.4	6.7	5.1	5.1	5.0	5.2	9.6 (0.4)
CSF	20.0	19.7	18.4	18.4	20.1	21.5	18.4	19.8	21.0	18.2	19.8	20.2	20.9	26.1	19.7 (1.4)

Volumes are in % of total intracranial volume. The control values are indicated as mean (SD). An age in red indicates that the patient was on treatment for at least 3 months. Yellow and blue indicate values larger or smaller than 2 SD (light colors) or 3 SD (dark colors) of the control range. y, years; m, months.

Table S3: Quantitative DTI measures of selected white matter structures.

	Subjects	Patient 1							Patient 2				Controls
	Age	1y8m	2y2m	2y7m	5y7m	6y5m	7y	8y	3y8m	4y3m	4y9m	5y9m	3y7m (14m)
FA	cerebral WM	0.22	0.21	0.16	0.11	0.13	0.16	0.17	0.09	0.09	0.10	0.15	0.25 (0.02)
	corpus callosum	0.39	0.39	0.42	0.34	0.36	0.33	0.31	0.36	0.32	0.31	0.27	0.51 (0.04)
	internal capsule	0.50	0.50	0.51	0.49	0.49	0.48	0.52	0.41	0.45	0.43	0.49	0.48 (0.03)
MD	cerebral WM	106.3	108.2	109.7	115.1	107.2	103.2	98.2	141.1	141.8	134.0	110.4	85.0 (3.5)
(10^{-5} mm^2/s)	corpus callosum	106.1	105.2	102.0	104.5	100.2	101.8	98.8	117.5	118.9	119.1	110.7	86.0 (4.4)
	internal capsule	96.4	96.4	94.5	85.4	83.4	82.5	83.1	99.3	91.9	93.4	89.2	81.2 (3.4)
RD	cerebral WM	93.6	96.0	100.5	107.8	99.8	94.7	89.0	132.6	133.2	126.6	100.8	73.5 (3.6)
(10^{-5} mm^2/s)	corpus callosum	85.5	84.2	78.6	84.3	81.3	83.5	82.7	94.0	97.9	98.2	93.0	59.7 (5.3)
	internal capsule	66.9	67.0	64.8	59.8	58.6	58.6	57.0	74.4	67.9	69.7	63.0	57.5 (4.1)
AD	cerebral WM	134.2	136.4	135.0	138.0	126.6	123.2	119.2	162.3	162.6	153.8	131.5	108.7 (3.3)
(10^{-5} mm^2/s)	corpus callosum	160.3	162.6	159.8	153.6	147.5	145.2	136.1	173.5	168.8	167.4	148.0	148.0 (6.1)
	internal capsule	159.3	159.5	158.8	139.4	136.4	135.0	138.0	149.7	145.0	144.6	143.2	130.7 (3.5)

The control values are indicated as mean (SD). An age in red indicates that the patient was on treatment for at least 3 months. Yellow and blue indicate values larger or smaller than 2 SD (light colors) or 3 SD (dark colors) of the control range. FA: fractional anisotropy, MD: mean diffusivity, RD: radial diffusivity, AD: axial diffusivity. y, years; m, months.

Table S4: Quantitative DTI measures of selected gray matter structures.

	Subjects	Patient 1							Patient 2				Controls
	Age	1y8m	2y2m	2y7m	5y7m	6y5m	7y	8m	3y8m	4y3m	4y9m	5y9m	3y7m (14m)
FA	cerebral cortex	0.11	0.10	0.10	0.07	0.08	0.08	0.09	0.06	0.06	0.06	0.07	0.11 (0.01)
	thalamus	0.24	0.24	0.25	0.23	0.22	0.23	0.23	0.23	0.22	0.22	0.23	0.22 (0.01)
	caudate nucleus	0.16	0.16	0.14	0.13	0.13	0.14	0.16	0.14	0.14	0.13	0.16	0.13 (0.01)
	putamen	0.18	0.17	0.15	0.13	0.16	0.17	0.18	0.13	0.13	0.15	0.20	0.15 (0.01)
	globus pallidus	0.22	0.21	0.20	0.20	0.15	0.12	0.14	0.17	0.18	0.14	0.19	0.19 (0.01)
MD	cerebral cortex	115.4	118.2	118.5	113.9	114.0	115.0	117.3	131.8	130.5	133.0	139.5	93.5 (4.7)
(10^{-5} mm^2/s)	thalamus	91.8	93.1	90.3	84.6	86.5	86.9	87.8	94.3	88.3	90.4	91.1	82.8 (3.2)
	caudate nucleus	93.3	92.7	93.2	91.6	91.7	91.0	91.3	100.9	98.8	103.5	101.3	83.7 (2.8)
	putamen	98.5	98.5	93.1	87.6	89.2	88.1	86.3	112.2	110.2	107.6	97.7	80.4 (2.9)
	globus pallidus	106.3	106.1	108.6	88.6	89.4	90.4	88.9	109.9	100.1	101.4	90.4	84.9 (3.5)
RD	cerebral cortex	109.0	111.8	112.1	109.5	109.2	110.0	111.6	127.4	126.4	128.7	133.8	88.0 (4.8)
(10^{-5} mm^2/s)	thalamus	79.1	80.2	78.1	74.6	76.2	75.9	77.1	82.8	78.5	79.8	81.2	74.1 (3.0)
	caudate nucleus	84.8	84.4	84.8	85.1	85.6	83.5	83.7	93.8	91.0	96.4	93.2	77.9 (3.0)
	putamen	89.6	89.9	86.2	81.6	80.7	80.0	77.9	105.7	104.0	99.5	88.4	74.4 (2.9)
	globus pallidus	93.4	94.3	97.1	79.3	82.3	84.1	82.3	100.7	90.5	92.8	82.2	76.2 (3.5)
AD	cerebral cortex	128.6	131.1	131.4	123.2	124.1	125.7	129.2	140.7	139.2	141.8	151.1	105.5 (4.7)
(10^{-5} mm^2/s)	thalamus	117.6	118.6	115.9	108.7	109.8	112.6	112.4	119.4	109.8	111.8	115.5	101.8 (3.6)
	caudate nucleus	109.7	108.4	107.6	105.2	105.2	104.1	106.1	115.7	116.0	119.0	118.7	94.8 (3.1)
	putamen	117.3	116.3	107.4	99.9	103.7	103.3	103.7	123.7	121.8	120.4	112.9	93.1 (2.9)
	globus pallidus	130.6	129.2	132.1	107.1	104.2	102.4	103.2	128.2	118.0	115.1	107.1	101.8 (3.9)

Control values are indicated as mean (SD). Age in red indicates that the patient was on treatment for at least 3 months. Yellow and blue indicate values larger or smaller than 2SD (light colors) or 3SD (dark colors) of the normal range. FA,: fractional anisotropy, MD: mean diffusivity, RD: radial diffusivity, AD: axial diffusivity. y, years; m, months.

Table S5: Laboratory findings for patients 1 and 2.

	**reference**	**patient 1**			**patient 2**		
		baseline	*after 6 months HCO_3^-*	*after 24 months HCO_3^-*	*baseline*	*after 4 months HCO_3^-*	*after 22 months HCO_3^-*
pH	7.36 – 7.44	7.37	**7.29 ↓**	**7.29↓**	**7.27 ↓**	7.40	7.34
pCO_2 (kPa)	4.7 – 6.0	**4.6 ↓**	5.5	**6.7 ↑**	**6.4 ↑**	**4.6**	**6.6 ↑**
HCO_3^- (mmol/L)	23 – 29	**19.6 ↓**	**19.4 ↓**	**23.5**	**21.3 ↓**	**20.6 ↓**	**25.9**
base excess (mmol/L)	-4 – 2	**-4.9 ↓**	**-6.8 ↓**	**-3.4**	**-5.7 ↓**	-3.5	**-0.4**
anion gap (mmol/L)[A]	7 – 18	13.4	12.6	8.5	11.7	12.4	6.1
K^+ (mmol/L)	3.5 – 5.1	4.0	3.8	4.3	3.6	4.3	4.0
creatinine (µmol/L)	31 – 68	31	27	27	31	29	36
eGFR (mL/min/1.73m^2)	> 80	> 90	>90	>90	>90	>90	>90

[A]Anion gap calculated based on serum concentration of Na^+, Cl^- and $HCO3^-$: anion gap = $[Na^+]$ - ($[Cl^-]$ + $[HCO_3^-]$). Updated reference range (7-18) based on Ayala-Lopez and Harb (2020).[18] Values in bold fall outside of the reference rang (↓ : lower; ↑ : higher).

Table S6: Ion substitution analysis of the p.(Ile805Thr)-NBCe1-B depolarizing leak.

	H_2O-injected			p.(Ile805Thr)-NBCe1-B injected		
10x change:	*↑K^+*	*↓Na^+ (↑$NMDG^+$)*	*↓Cl^- (↑Gluconate)*	*↑K^+*	*↓Na^+ (↑$NMDG^+$)*	*↓Cl^- (↑Gluconate)*
ΔV_m (mV)	12 ± 1	−4 ± 1	6 ± 1	1 ± 1	−8 ± 1	8 ± 1
n	8	8	8	8	8	8
p vs H_2O				< 0.01	0.01	0.07

Data are presented as mean ± SEM. *p* indicates results from a one-tailed t-test.

Text belonging to Table S6:

To interrogate the ionic permeability underlying the depolarizing leak, we quantified membrane potential deflections (ΔV_m) in H_2O-injected and p.(Ile805Thr)-NBCe1-B-expressing oocytes in response to defined extracellular ion substitutions. Specifically, extracellular K^+ was increased tenfold, or extracellular Na^+ or Cl^- was reduced tenfold by 90% substitution with $NMDG^+$ or gluconate, respectively. Resulting ΔV_m values are summarized in Table S6. Resting membrane potentials were −39 ± 1 mV for H_2O-injected oocytes (n = 24) and −23 ± 1 mV for p.(Ile805Thr)-expressing oocytes (n = 24).

The magnitude of ΔV_m is a proxy for the relative permeability of the cell membrane to an ion. For a cell expressing a perfectly selective ion channel, a 10-fold change in the concentration of that ion ought to cause a deflection of 58 mV. For a cell expressing a mixture of ionic permeabilities, the sum of the deflections to individually imposed 10-fold changes in those permeable ions ought to total 58 mV.

Expression of p.(Ile805Thr)-NBCe1-B markedly reduced the contribution of endogenous K^+ conductance to membrane potential, consistent with the leak overwhelming endogenous oocyte K^+ permeability. In contrast, relative Cl^- permeability was largely preserved, whereas apparent Na^+ permeability was increased compared to control oocytes. The summed ΔV_m responses did not approach the theoretical 58 mV expected for a single highly selective permeation pathway, reflecting the substantial permeability of Xenopus oocytes to the substituting ions $NMDG^+$ and gluconate.[19]

To assess whether the observed responses could be reconciled by changes in relative ionic permeabilities, we implemented the Goldman-Hodgkin Katz equation with the standard Nernstian relationship for K^+ ($58 * \log_{10}[K^+]_o/[K^+]_i$) and a modified Nernstian relationship for Na^+ and Cl^- to account for pathway permeability to the substituting ion,[20] such that:

$$\Delta V_m = P_{cation}.\Delta E_{cation} + P_K.\Delta E_K + P_{anion}.\Delta E_{anion}$$

For changes in $[K^+]_o$, which are made without substitution, the influence of K^+ on V_m is calculated as:

$$\Delta E_K = 58 \times log_{10}\left(\frac{[K^+]_{o,final}}{[K^+]_{o,initial}}\right)$$

For changes in $[Cl^-]_o$, which are made by reciprocal substitution with gluconate, the influence of these anions on V_m is calculated as:

$$\Delta E_{anion} = 58 \times log_{10}\left(\frac{[Cl^-]_{o,initial}}{[Cl^-]_{o,final} + \left({P_{gluconate}}/{P_{Cl}} \times [Gluconate]_{o,final}\right)}\right)$$

For changes in $[Na^+]_o$, which are made by reciprocal substitution with $NMDG^+$, the influence of these cations on V_m is calculated as:

$$\Delta E_{cation} = -58 \times log_{10}\left(\frac{[Na^+]_{o,initial}}{[Na^+]_{o,final} + \left({}^{P_{NMDG}}/_{P_{Na}} \times [NMDG^+]_{o,final}\right)}\right)$$

There are presumably many solutions to this set of equations. As an example, we can approximate our observations of H_2O-injected cells if we implement a poorly selective cation permeability (P_{cation}), a poorly selective anion permeability (P_{anion}) and a permeability to potassium (P_K) with the following parameters:

P_{cation} = 0.35, P_{NMDG}/P_{Na} = 0.6

P_K = 0.2

P_{anion} = 0.45, $P_{gluconate}/P_{Cl}$ = 0.55.

These parameters allow for a 12 mV deflection to K increase, a 4 mV deflection to Na^+ replacement, and a 6 mV deflection to Cl^- replacement.

Only two major modifications to these parameters are required to approximate our observations of p.(Ile805Thr)-NBCe1-B-expressing oocytes: 1. Resetting P_K to 0, with a relative rebalancing of P_{cation} to 0.44 and P_{anion} to 0.56, and 2. A decrease in P_{NMDG}/P_{Na} from 0.6 to 0.45, reflecting an enrichment of Na^+-selectivity of cationic pathways. A small incremental change in $P_{gluconate}/P_{Cl}$ to 0.54 is also necessary for complete alignment.

These parameters allow for V_m to be K^+ independent, an enhanced 8 mV deflection to Na^+ replacement, and a preserved 8 mV deflection to Cl^- replacement.

In summary, ion substitution experiments combined with permeability calculations indicate that the depolarizing leak associated with p.(Ile805Thr)-NBCe1-B is sufficient to overwhelm endogenous oocyte K^+ conductance, preserves an anion permeability comparable to the endogenous Cl^-/gluconate conductance ratio, and displays increased Na^+ selectivity relative to endogenous cation permeability. These properties are incompatible with formation of a highly selective ion channel and instead support the presence of a mixed, weakly selective depolarizing conductance.

References

1. Wolf NI, Salomons GS, Rodenburg RJ, et al. Mutations in RARS cause hypomyelination. Ann Neurol. 2014 Jul;76(1):134–9.
2. van Rappard DF, Konigs M, Steenweg ME, et al. Diffusion tensor imaging in metachromatic leukodystrophy. J Neurol. 2018 Mar;265(3):659–68.
3. Stellingwerff MD, Al-Saady ML, Chan KS, et al. Applicability of multiple quantitative magnetic resonance methods in genetic brain white matter disorders. J Neuroimaging. 2024 Jan–Feb;34(1):61–77.
4. Billot B, Greve DN, Puonti O, et al. SynthSeg: Segmentation of brain MRI scans of any contrast and resolution without retraining. Med Image Anal. 2023 May;86:102789.
5. Andersson JL, Skare S, Ashburner J. How to correct susceptibility distortions in spin-echo echo-planar images: application to diffusion tensor imaging. Neuroimage. 2003 Oct;20(2):870–88.

6. Andersson JLR, Sotiropoulos SN. An integrated approach to correction for off-resonance effects and subject movement in diffusion MR imaging. Neuroimage. 2016 Jan 15;125:1063–78.
7. Avants BB, Tustison NJ, Song G, Cook PA, Klein A, Gee JC. A reproducible evaluation of ANTs similarity metric performance in brain image registration. Neuroimage. 2011 Feb 1;54(3):2033–44.
8. Schindelin J, Arganda-Carreras I, Frise E, et al. Fiji: an open-source platform for biological-image analysis. Nat Methods. 2012 Jun 28;9(7):676–82.
9. Suzuki M, Van Paesschen W, Stalmans I, et al. Defective membrane expression of the Na(+)-HCO(3)(-) cotransporter NBCe1 is associated with familial migraine. Proc Natl Acad Sci U S A. 2010 Sep 7;107(36):15963–8.
10. Zhang Y, Werling U, Edelmann W. Seamless Ligation Cloning Extract (SLiCE) cloning method. Methods Mol Biol. 2014;1116:235–44.
11. Lee SK, Boron WF, Parker MD. Relief of autoinhibition of the electrogenic Na-HCO(3) [corrected] cotransporter NBCe1-B: role of IRBIT vs.amino-terminal truncation. Am J Physiol Cell Physiol. 2012 Feb 1;302(3):C518–26.
12. Brady CT, Marshall A, Zhang C, Parker MD. NBCe1-B/C-knockout mice exhibit an impaired respiratory response and an enhanced renal response to metabolic acidosis. Front Physiol. 2023;14:1201034.
13. Abramson J, Adler J, Dunger J, et al. Accurate structure prediction of biomolecular interactions with AlphaFold 3. Nature. 2024 Jun;630(8016):493–500.
14. Smart OS, Goodfellow JM, Wallace BA. The pore dimensions of gramicidin A. Biophys J. 1993 Dec;65(6):2455–60.
15. Sjostedt E, Zhong W, Fagerberg L, et al. An atlas of the protein-coding genes in the human, pig, and mouse brain. Science. 2020 Mar 6;367(6482).
16. Uhlen M, Fagerberg L, Hallstrom BM, et al. Tissue-based map of the human proteome. Science. 2015 Jan 23;347(6220):1260419.
17. Zhang Y, Sloan SA, Clarke LE, et al. Purification and Characterization of Progenitor and Mature Human Astrocytes Reveals Transcriptional and Functional Differences with Mouse. Neuron. 2016 Jan 6;89(1):37–53.
18. Ayala-Lopez N, Harb R. Interpreting Anion Gap Values in Adult and Pediatric Patients: Examining the Reference Interval. J Appl Lab Med. 2020 Jan 1;5(1):126–35.
19. Costa PF, Emilio MG, Fernandes PL, Ferreira HG, Ferreira KG. Determination of ionic permeability coefficients of the plasma membrane of Xenopus laevis oocytes under voltage clamp. J Physiol. 1989 Jun;413:199–211.
20. Parker MD, Young MT, Daly CM, Meech RW, Boron WF, Tanner MJ. A conductive pathway generated from fragments of the human red cell anion exchanger AE1. J Physiol. 2007 May 15;581(Pt 1):33–50.
